# Natural variation of *OsGluA2* is involved in grain protein content regulation in rice

**DOI:** 10.1038/s41467-019-09919-y

**Published:** 2019-04-26

**Authors:** Yihao Yang, Min Guo, Shengyuan Sun, Yelu Zou, Shuangyi Yin, Yannan Liu, Shuzhu Tang, Minghong Gu, Zefeng Yang, Changjie Yan

**Affiliations:** 1 Jiangsu Key Laboratory of Crop Genetics and Physiology/Key Laboratory of Plant Functional Genomics of the Ministry of Education/Jiangsu Key Laboratory of Crop Genomics and Molecular Breeding, Yangzhou University, 225009 Yangzhou, China; 2 Jiangsu Co-Innovation Center for Modern Production Technology of Grain Crops, Yangzhou University, 225009 Yangzhou, China; 3 Agricultural College, Yangzhou University, 225009 Yangzhou, China

**Keywords:** Agricultural genetics, Agricultural genetics, Natural variation in plants

## Abstract

Grain protein content (GPC) affects rice nutrition quality. Here, we identify two stable quantitative trait loci (QTLs), *qGPC-1* and *qGPC-10*, controlling GPC in a mapping population derived from *indica* and *japonica* cultivars crossing. Map-based cloning reveals that *OsGluA2*, encoding a glutelin type-A2 precursor, is the candidate gene underlying *qGPC-10*. It functions as a positive regulator of GPC and has a pleiotropic effect on rice grain quality. One SNP located in *OsGluA2* promoter region is associated with its transcript expression level and GPC diversity. Polymorphisms of this nucleotide can divide all haplotypes into low (*OsGluA2*^*LET*^) and high (*OsGluA2*^*HET*^) expression types. Population genetic and evolutionary analyses reveal that *OsGluA2*^*LET*^, mainly present in *japonica* accessions, originates from wild rice. However, *OsGluA2*^*HET*^, the dominant type in *indica*, is acquired through mutation of *OsGluA2*^*LET*^. Our results shed light on the understanding of natural variations of GPC between *indica* and *japonica* subspecies.

## Abstract (web summary)

Grain protein content determines rice nutrition quality. Here, the authors show that a single nucleotide polymorphism in the promoter region of OsGluA2, encoding a glutelin type-A2 precursor, is responsible for glutelin content difference between the *indica* and *japonica* rice subspecies.

## Introduction

Rice (*Oryza sativa* L.) is the most important human food crop in the world, providing over 21% of the calorific needs for the world’s population and up to 76% of the calorific intake for the South East Asian^1^. With the improvement of people’s living standards, rice consumers are paying much attention to good grain quality. Rice grain quality is composed of four aspects, grain appearance, milling quality, nutrition quality, and cooking and eating quality^2,3^. Of which, nutrition quality as well as cooking and eating quality are two most important indices that are widely concerned by the consumers. Three major components in endosperm, i.e. starch (70–80%), protein (7–10%), and lipids (~1%), predominantly determine the nutrition quality and cooking and eating quality^4^. Therefore, unraveling the genetic basis underlying the synthesis of starch, protein, and lipids is a prerequisite for the improvement of rice grain quality. In recent decades, great achievements have been made in the dissection of starch synthesis pathway and the genetic network controlling starch variations^5–8^. However, the genetic basis for grain protein content (GPC), another important factor affecting rice nutrition quality as well as eating and cooking quality remains largely unclear.

Rice grain protein consists of two categories, functional protein (~10%) and seed storage protein (SSP, ~90%). According to the solubility-linked physical properties, SSPs are classified as four fractions: albumins, globulins, prolamins, and glutelins^9,10^. Among them, glutelin is the most abundant one, which comprises about 60–80% of the total SSPs^11^. The nutritional value of rice glutelin is superior to other rice storage proteins due to its higher lysine content and greater digestibility by the humans^12^. Therefore, any large change of glutelin content will certainly affect the grain nutrition quality. Glutelins can be further divided into four groups (GluA, GluB, GluC, and GluD) based on their amino acid sequence similarity, and synthesized as a 57 kDa precursor and then cleaved into a 37–39 kDa acidic subunit and a 22–23 kDa basic subunit in the cytoplasm^13^.

Asian cultivated rice is generally divided into two main subspecies, *indica* and *japonica*. Investigations have revealed that both *indica* and *japonica* cultivars have substantial variation in GPC with a range from 4.9% to 19.3% and from 5.9% to 16.5%, respectively, and that the mean of GPC in *indica* is generally higher than that of *japonica*^14,15^. The difference of GPC between *indica* and *japonica* might be due to their different genetic architectures^16,17^. However, the mechanism underlying the GPC differences remains largely unclear.

Significant efforts have been taken toward dissecting the genetic mechanism of rice GPC. Consequently, many genes (regulators) affecting GPC have been isolated from various mutants in rice^11,18–25^, along with hundreds of QTLs have been reported^26–34^. However, GPC belongs to a typical quantitative trait with complex genetic structure and is susceptible to environmental factors, especially to nitrogen fertilization in the late growth stages. Thus, the QTLs for rice GPC detected using different genetic populations are frequently inconsistent, and few of them can be repeatedly detected. To date, only one QTL (*qPC1*), on the long arm of chromosome 1, has been cloned. *qPC1* encodes a putative amino acid transporter *OsAAP6* and functions as a positive regulator of rice GPC^35^. Beyond that, little progress has been achieved in the breeding improvement of rice GPC due to lacking of gene targets.

In this study, we illustrate the putative genetic mechanism of rice GPC variation through QTL mapping, gene cloning, and functional detection, and demonstrate that glutelin content is the major contributor to the variation of rice GPC between *indica* and *japonica* subspecies. Two environmentally stable QTLs for GPC, *qGPC-1* and *qGPC-10*, are identified. *qGPC-10*, encoding a glutelin type-A2 precursor OsGluA2, is isolated through map-based cloning. We reveal that *OsGluA2* functions as a positive regulator of GPC through complementation, knockout (CRISPR-Cas9), and natural variation analyses. Our evolutionary and population genetic analyses show that *OsGluA2* significantly contributes to the genetic difference between *indica* and *japonica* subspecies.

## Results

### Glutelin content contributes largely to GPC variation

**Fig. 1:**
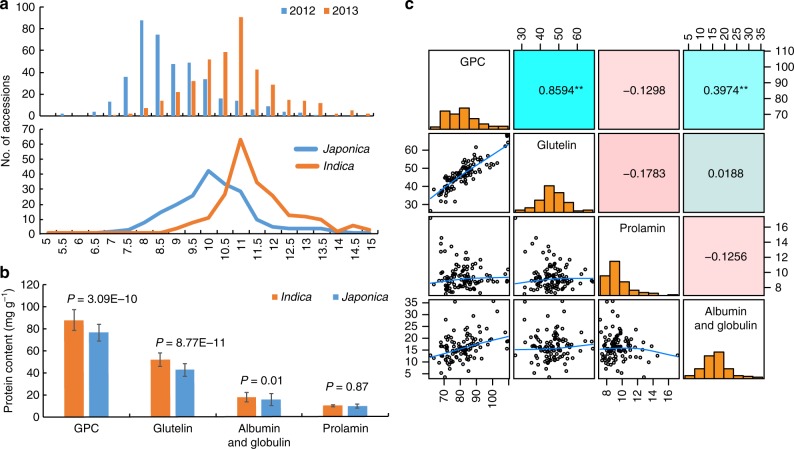
*Indica* varieties show higher GPC and glutelin contents than *japonica* varieties. **a** The distribution of GPC among 402 accessions in 2012 and 2013, and the distribution of GPC within the subspecies *indica* and *japonica* in 2013. **b** Comparison of GPC and storage protein fraction between the 43 conventional *indica* varieties and 60 *japonica* varieties. Error bars, s.d. *P*-values were calculated by independent-samples *t*-test. **c** The pairwise correlation analysis among four total kinds of protein content. Upper diagonal: Pearson correlation coefficients between every two traits; lower diagonal: Scatter plots of correlations between every two traits. Double asterisks (**) indicates significance level at *P* < 0.01

We firstly surveyed GPC in a core collection of 402 rice accessions, including 205 *indica* and 197 *japonica* cultivars, in two environmental conditions^36,37^. The results showed that GPC differs tremendously among rice cultivars, ranging from 5.33% to 14.83%, 81.5% of cultivars concentrated in the range of 7.5–11.5% (Fig. 1a). Moreover, as expected, environmental factor has a significant effect on GPC as the mean of GPC in 2013 (10.56 ± 0.07%) is significantly higher than that in 2012 (8.66 ± 0.06%) (Supplementary Table 1).

GPC variation was further analyzed in *indica* and *japonica* subspecies, respectively. The results indicated that there is a significant difference between the two species in both two environmental conditions. The GPC of *indica* cultivars is 9.29 ± 0.09%, while 7.99 ± 0.07% in *japonica* in 2012. The same tendency was also found in 2013; the GPC are 11.19 ± 0.08% and 9.91 ± 0.08% for *indica* and *japonica*, respectively (Supplementary Table 1). Using the values of $\bar x$ ± s.d. as quantile, all the rice accessions were classified into three types, high, medium and low types. We noticed that the frequency distributions in the two subspecies are unbalanced in three types (*χ*^*2*^ = 6.474, *P* = 0.039). For *indica* subspecies, it possesses a higher proportion of high GPC cultivars;inversely, higher proportion of low GPC accessions occurs in *japonica* subspecies (Supplementary Table 2). Thus, it was concluded that *indica* cultivars exhibited higher GPC than *japonica*, consistent with previous findings^14,15^.

To address why the GPC in *indica* subspecies is generally higher than that in *japonica*, 43 conventional *indica* and 60 *japonica* cultivars from 402 accessions with similar heading date and plant height were chosen to establish a subpopulation subject to further assay of four fractions of SSP by the Kjeldahl method^28^. Statistical analysis revealed that glutelin and albumin + globulin contents show significant difference between the two subspecies, explaining 73.9% and 15.8% of the total variance of GPC, while no significant differences were detected for prolamin content in the test population (Fig. 1b, c). Thus, it is very clear that glutelin content variation acts as a major contributor to the GPC variation between *indica* and *japonica* subspecies, suggesting that glutelin content could be directly used as a target for rice nutrition quality improvement.

### Identification of two major QTLs for GPC variation

**Fig. 2:**
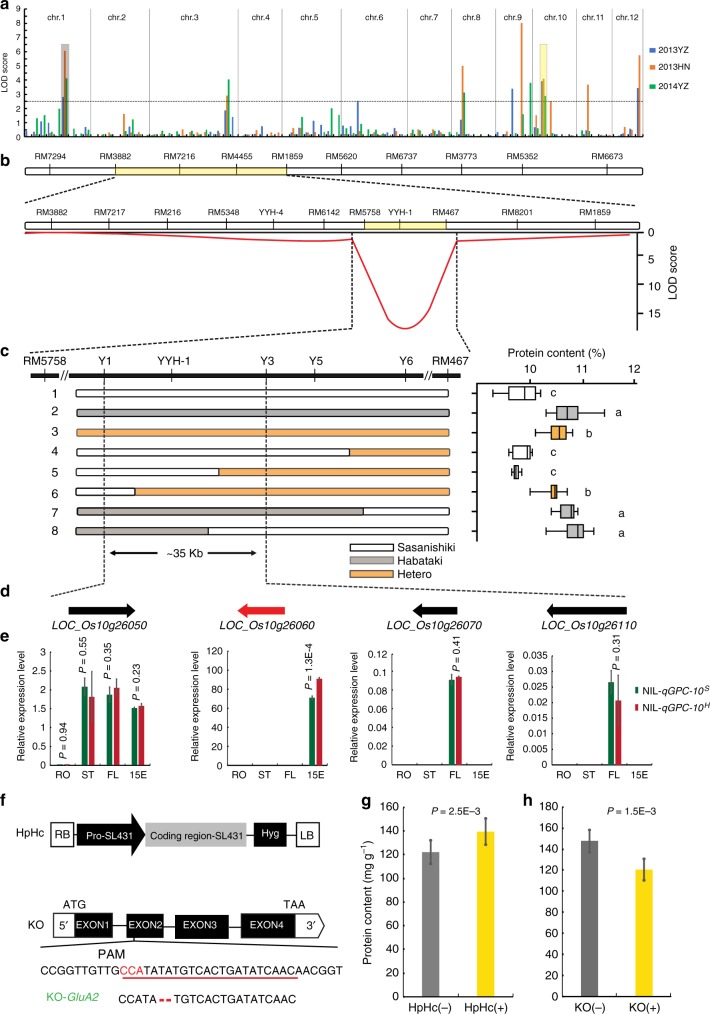
Map-based cloning of the *qGPC-10*. **a** Graph for QTL mapping results of GPC with CSSL population in three different environments. **b** Location of the *qGPC-10* on the chromosomal 10 and *qGPC-10* was delimited to the region governed by RM5758 and RM467 by using an F_2_ population. **c** Fine mapping of *qGPC-10*. Left, high-resolution mapping. Right, phenotypes of recombinants. Boxes represent the median values and the first and third quartiles; whiskers represent the minimum and maximum values. The presence of the same lowercase letter denotes a non-significant difference between them (*P* > 0.05). *P*-values were calculated by one-way ANOVA. **d** The four predicted ORFs. **e** The expression patterns of the four predicted ORFs by qRT-PCR analysis. Tissues: RO, ST and FL: root, stem, and flag leaf at heading stage with 1 cm panicle out of the leaf sheath; 15E: endosperms at 15 DAF. Error bars, s.d., *n* = 3. *P*-values were calculated by independent-samples *t*-test. **f** Schematic of vectors for transgenic analysis. HpHc, complementation vector; KO, knockout vector, the target site is underlined and the PAM is highlighted in red. The deleted sequences are shown by red hyphens. **g**, **h** GPC of transgenic plants in T_1_. (−) and (+) indicate transgene-negative and transgene-positive plants. Error bars, s.d., *n* = 15 in HpHc(−) and HpHc(+), and *n* = 8 in KO(−) and KO(+). *P*-values were calculated by independent-samples *t*-test. Source data of Figs. 2a–c and 2e–h are provided as a Source Data file

To understand the genetic mechanisms of GPC in rice, a chromosomal segment substitution line (CSSL) population derived from a cross of an *indica* cultivar Habataki and a *japonica* cultivar Sasanishiki was employed for QTL mapping (Supplementary Table 3). In this population, a total of 18 QTLs for GPC were identified across three environmental conditions with a threshold of LOD ≥ 2.5. Among them, *qGPC-1* and *qGPC-10* were repeatedly identified under all three environmental conditions, while *qGPC-3, qGPC-8*, and *qGPC-12* were detected in two environmental conditions, and others can only be identified in one environmental condition, suggesting they might be sensitive to environmental factors (Fig. 2a and Supplementary Table 4). The alleles from Habataki on *qGPC-3, qGPC-8, qGPC-10*, and *qGPC-12* can increase GPC, while the allele of *qGPC-1* will decrease GPC.

### Delimiting *qGPC-10* to a 35-kb long region

Because *qGPC-10* is a stably inherited QTL with large effect for GPC, map-based cloning has been carried out for *qGPC-10*. According to the preliminary mapping results, the CSSL line SL431 harboring *qGPC-10* was selected as parent to cross with Sasanishiki to develop an F_2_ population for fine mapping (Supplementary Fig. 1a, b). The grain protein profiles of two parents were analyzed by using SDS-PAGE analysis. The densitometry analysis indicates that SL431 has higher accumulation of proglutelins, acidic and basic subunits, as well as prolamins than those in Sasanishiki (Supplementary Fig. 1c). This observation was further confirmed by the results of protein fraction analysis (Supplementary Fig. 1d). Unexpectedly, a dramatic segregation of GPC in F_2_ population containing 2085 plants was observed, ranging from 8.4% to 13.4% with normal distribution (*P* = 0.113 > 0.05, K–S test), even though the genetic background of SL431 is similar to Sasanishiki (Supplementary Fig. 2a–c). In order to eliminate the environmental interference, a total of 790 plants with extreme phenotypes, namely, the GPC ranging from 8.4% to 9.5% and from 11.5% to 13.4%, respectively, were selected to generate a subpopulation for fine mapping. As a result, *qGPC-10* was successfully delimited to the interval between the marker RM5758 and RM467, the genetic distance was about 3.83 cM (Fig. 2b).

To further narrow down the *qGPC-10* anchoring interval, we selected the F_2_ individuals with the heterozygous genotypes on RM467 and RM5758 to generate an F_3_ population. The 1085 F_3_ individuals with extreme phenotypes (GPCs ranging from 8.8% to 10% and 10.5% to 11.4%) were selected for fine mapping. A total of 97 recombinants in the interval between RM467 and RM5758 was found based on their genotypes, which were divided into eight types. Multiple comparison analysis showed that the eight types could be classified into three groups based on their GPCs (Fig. 2c and Supplementary Table 5). This result clearly indicates that *qGPC-10* co-segregates with marker YYH-1, locating between marker Y1 and Y3, and the physical distance between them was around 35 kb. Interestingly, the fact that the GPC in heterozygous plants is significantly higher than in Sasanishiki homozygotes but lower than in Habataki homozygotes indicates that the *qGPC-10*^*H*^ allele (allele from Habataki) is partially dominant.

According to the information from Rice Genome Annotation Project, four predicted genes are present in this region (Fig. 2d and Supplementary Table 6), namely, *LOC_Os10g26050*, *LOC_Os10g26060*, *LOC_Os10g26070,* and *LOC_Os10g26110*. We measured the expression levels of these genes in different organs through quantitative reverse transcription-PCR (qRT-PCR) (Fig. 2e). Only *LOC_Os10g26060*, putatively encoding a precursor of glutelin, differentially expresses in endosperms between NILs, being about 1.3-fold higher in NIL-*qGPC-10*^*H*^ (high GPC) than in NIL-*qGPC-10*^*S*^ (low GPC) (*P* = 3.0 × 10^–4^, independent-samples *t*-test). Therefore, we deduced that the gene *LOC_Os10g26060* encoding glutelin type-A2 precursor (referred to hereafter as *OsGluA2*) was responsible to *qGPC-10*.

### *OsGluA2* is the candidate gene underlying *qGPC-10*

In order to confirm the gene *OsGluA2* is responsible to *qGPC-10*, two constructs were generated and transformed to the corresponding genotypes through *agrobacterium*-mediated transformation (Fig. 2f). Firstly, a complementary construct (HpHc) containing ~2 kb promoter fragment with full length of *OsGluA2* from SL431 was generated and introduced into the parent Sasanishiki. Compared to transgene-negative plants, transgene-positive plants in T_1_ progeny for HpHc(+) showed a significant (*P* = 2.5 × 10^–3^, independent-samples *t*-test) increase in the GPC (Fig. 2g). Secondly, one CRISPR-Cas9 system construct-expressing guide RNAs targeting the second exon of *OsGluA2* was developed and used to transform into SL431. We screened for the KO-*OsGluA2* with two-base deletion in the second exon predicted to produce truncated polypeptides with 187 amino acids (Supplementary Fig. 3b–d). As expected, grains produced from homozygous KO-*OsGluA2* plants showed a lower GPC phenotype (Fig. 2h). Meanwhile, there were no significant differences between the NILs and transgenic plants in other agronomic traits and yield components (Supplementary Fig. 3a and Supplementary Table 7). All these evidences confirm that *OsGluA2* is responsible to *qGPC-10*.

### Higher expression level of *OsGluA2* promotes GPC

**Fig. 3:**
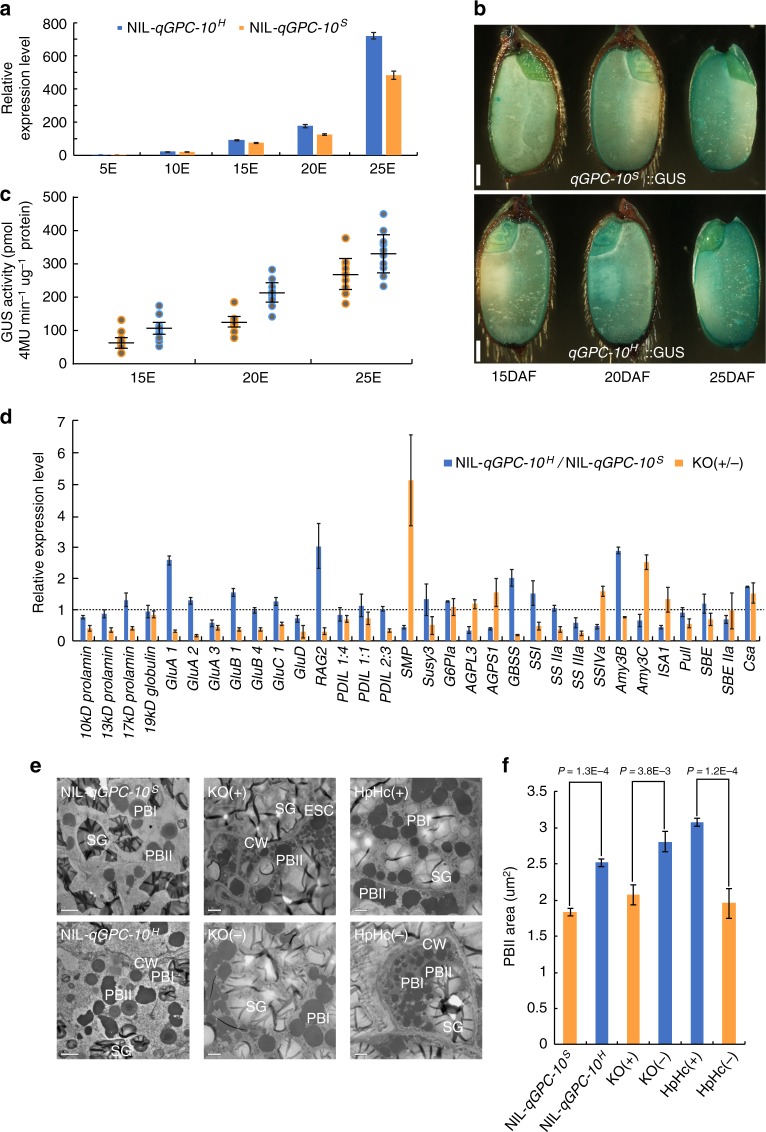
The expression patterns of *OsGluA2* and genes involved in storage materials. **a** The expression patterns of *OsGluA2* was determined by qRT-PCR. 5E, 10E, 15E, 20E, and 25E: endosperms at 5, 10, 15, 20, and 25 DAF. *n* = 3. **b** GUS assays in transgenic rice carrying *qGPC-10*^*S*^::GUS and *qGPC-10*^*H*^::GUS at 15, 20, and 25 DAF. Scale bar = 1 mm. **c** GUS activity in maturing seeds at 15, 20, and 25 DAF. GUS activity is expressed as pmol 4MU min^−1^ μg^−1^ protein. *n* = 10. **d** Expression levels of key genes involved in synthesis and storage of endosperm components in T_2_ transgenic plants and NILs. Transgene-positive plants of *OsGluA2* and NIL-*qGPC-10*^*H*^ are shown relative to transgene-negative plants and NIL-*qGPC-10*^*S*^, respectively, set as 1. Error bars, s.d., *n* = 3. **e** Ultrastructure of cells in developing endosperm of NILs and transgenic plants in 15 DAF. PB I and PB II protein bodies I and II, SG starch granule, ECS extracellular space, CW cell wall. Scale bars = 2 μm. **f** Quantitative comparison of PB II area in NILs and transgenic plants. *P*-values were calculated by independent-samples *t*-test. Error bars, s.d., *n* = 3. Source data of Figs. 3a, 3c, 3d and 3f are provided as a Source Data file

In order to illustrate how *OsGluA2* regulates GPC, qRT-PCR was used to investigate the expression patterns of *OsGluA2* between NILs (NIL-*qGPC-10*^*H*^ and NIL-*qGPC-10*^*S*^) (Fig. 3a). *OsGluA2* transcripts were abundant in the endosperms at 15–25 days after flowering (DAF), similar to the previous results^28^. Furthermore, significant differences of expression level were observed at 15 DAF, higher in NIL-*qGPC-10*^*H*^ than in NIL-*qGPC-10*^*S*^ during the endosperm development. The different expression level of *OsGluA2* in NILs implies that the sequence variations in promoter region may affect the expression level of *OsGluA2*, and thus resulting in the divers GPCs.

The expression pattern of *OsGluA2* was further analyzed by using transgenic plants carrying *qGPC-10*^*S*^::GUS (*OsGluA2* promoter from Sasanishiki) and *qGPC-10*^*H*^::GUS (*OsGluA2* promoter from SL431), respectively. The GUS staining in the endosperm gradually went darker along with grain filling, and the GUS activity of *qGPC-10*^*H*^::GUS was higher than that of *qGPC-10*^*S*^::GUS at 15, 20, and 25 DAF, respectively, consistent with the result obtained by qRT-PCR (Fig. 3b, c).

### *OsGluA2* has pleiotropic effects on rice grain quality

To explore the genetic effect of *OsGluA2* on rice grain quality, the NILs and transgenic plants were investigated for GPC, SSP fractions, as well as starch content and physic-chemical properties (Table 1). The GPC, as well as glutelin, albumin, and prolamin contents in NIL-*qGPC-10*^*H*^ are significantly higher than NIL-*qGPC-10*^*S*^, with an exception of globulin contents. Similar results were also observed in complementation (HpHc) and knockout (KO-*OsGluA2*) transgenic progenies. These results clearly indicate that *OsGluA2* has a noticeable effect on GPC and most of the SSP fractions, with the largest effect on glutelin variations (*P* = 2.5–8.4 × 10^–4^, independent-samples *t*-test). Hence, it is considered that the change in glutelin quantity is the major contributor to the total GPC variations in NILs and transgenic plants. Additionally, the starch contents and amylose contents (AC) were slightly changed subjecting to the genetic backgrounds. However, the gel consistencies (GCs) were altered significantly. When the GPC increases, the GC dramatically decreases in NILs (*P* = 3.7 × 10^–4^, independent-samples *t*-test) and complementation test (*P* = 4.8 × 10^–3^, independent-samples *t*-test). Similarly, when GPC in KO(+) plants decreases, GC increases (*P* = 7.2 × 10^–3^, independent-samples *t*-test). The fact that the GPC is negatively correlated with GC implies that GPC might affect rice eating and cooking quality as well.

**Table 1:** Grain quality traits in NILs and transgenic plants

Lines	*n*	Protein content (mg g^−1^)	Albumins content (mg g^−1^)	Globulins content (mg g^−1^)	Prolamins content (mg g^−1^)	Glutelins content (mg g^−1^)	AC (%)	GC (mm)	Starch content (%)
NIL-*qGPC-10*^*S*^	20	131.3 ± 4.5	19.5 ± 0.8	20.6 ± 0.1	10.1 ± 0.6	68.4 ± 3.4	15.9 ± 0.4	93.7 ± 4.5	88.5 ± 0.7
NIL-*qGPC-10*^*H*^	20	161.4 ± 6.0	23.7 ± 0.9	20.5 ± 0.1	12.5 ± 0.4	85.5 ± 3.2	14.9 ± 0.3	70.0 ± 4.1	86.1 ± 0.5
*P*-value		2.9 × 10^−4^	2.5 × 10^−3^	9.7 × 10^−2^	1.0 × 10^−3^	8.4 × 10^−4^	6.7 × 10^−2^	3.7 × 10^−4^	5.1 × 10^−2^
HpHc (−)	20	129.8 ± 3.9	13.2 ± 1.1	19.2 ± 0.7	12.4 ± 1.6	69.9 ± 1.9	15.7 ± 0.3	94.4 ± 3.2	87.9 ± 0.3
HpHc (+)	20	148.1 ± 5.3	21.8 ± 1.4	20.8 ± 0.6	19.2 ± 1.5	80.1 ± 1.6	14.8 ± 0.3	80.6 ± 3.3	85.6 ± 0.4
*P*-value		7.9 × 10^−3^	2.4 × 10^−5^	9.3 × 10^−2^	4.0 × 10^−3^	2.5 × 10^−4^	5.2 × 10^−2^	4.8 × 10^−3^	1.2 × 10^−2^
KO (−)	20	153.9 ± 3.7	20.3 ± 0.8	21.8 ± 0.2	11.3 ± 0.7	83.7 ± 2.7	14.9 ± 0.4	72.3 ± 4.6	86.5 ± 0.3
KO (+)	20	136.8 ± 3.0	16.6 ± 1.1	21.4 ± 0.3	9.0 ± 0.3	70.1 ± 2.3	16.4 ± 0.3	91.2 ± 4.8	88.8 ± 0.3
*P*-value		9.5 × 10^−4^	7.7 × 10^−3^	2.4 × 10^−1^	4.0 × 10^−3^	4.8 × 10^−4^	3.5 × 10^−3^	7.2 × 10^−3^	7.2 × 10^−3^

*AC* amylose content, *GC* gel consistency. At least 300 grains of each plant were harvested. All data are given as means ± s.e. (*n* = 3 in starch content). *P*-values were calculated by *t*-test. Source data of Table 1 are provided as a Source Data file

To determine whether the alteration in the accumulation of SSPs and starch are due to the transcriptional changes of other genes related to grain storage materials, we examined the transcription levels of 32 genes, including 16 hallmark genes involved in SSP synthesis and 16 genes related to starch metabolism at 15 DAF (Fig. 3d). In general, the expression of genes encoding glutelins (*GluA1, GluA2, GluB1, GluC*, and *RAG2*) and prolamins (*17-kD prolamins*) are up-regulated at various extent in NIL-*qGPC-10*^*H*^ when compared with NIL-*qGPC-10*^*S*^. Similarly, these genes are noticeably down-regulated in KO-*OsGluA2* plants. At the same time, the transcriptional level of genes related to starch metabolism also changed to various extents. These results strongly suggest that *OsGluA2* has significant associating effect on the expression of a large portion of other genes participating in grain storage materials metabolism in rice developing grains.

Moreover, the total amino acid contents of grains were also measured both in the NILs and transgenic plants (Supplementary Fig. 4). Compared with the corresponding levels in NIL-*qGPC-10*^*S*^ and transgene-negative HpHc(−) plants, the amounts of aspartic acid, threonine, glutamic acid + glutamine, isoleucine, lysine, arginine and proline amino acids and total content of amino acids were significantly increased in NIL-*qGPC-10*^*H*^ and transgene-positive HpHc(+) plants, respectively, whereas the reverse was true for the KO-*OsGluA2*. These results suggest that *OsGluA2* enhances GPC by increasing the grain storage protein content and the total amount of amino acids, and thus improves nutritional quality.

### GPC enriched grains have more and larger protein body II

We anatomically determined the effect of *OsGluA2* on protein body (PB) formation by transmission electronic microscope (Fig. 3e). In the developing endosperms at 15 DAF, two types of PBs can be well discerned in ultrathin sections, including prolamins-containing PB I and glutelin/globulin-containing PB II. In the developing endosperms of NIL-*qGPC-10*^*H*^ and HpHc(+), the size (section areas) of PB II were more expanded, whereas the opposite was true for KO(+), comparing to the control plants (Fig. 3f). These results indicate that *OsGluA2* has enhanced the total amount of glutelin content, resulting in the size enlargement and increased number of PB IIs.

### One SNP is associated with transcriptional level and GPC

**Fig. 4:**
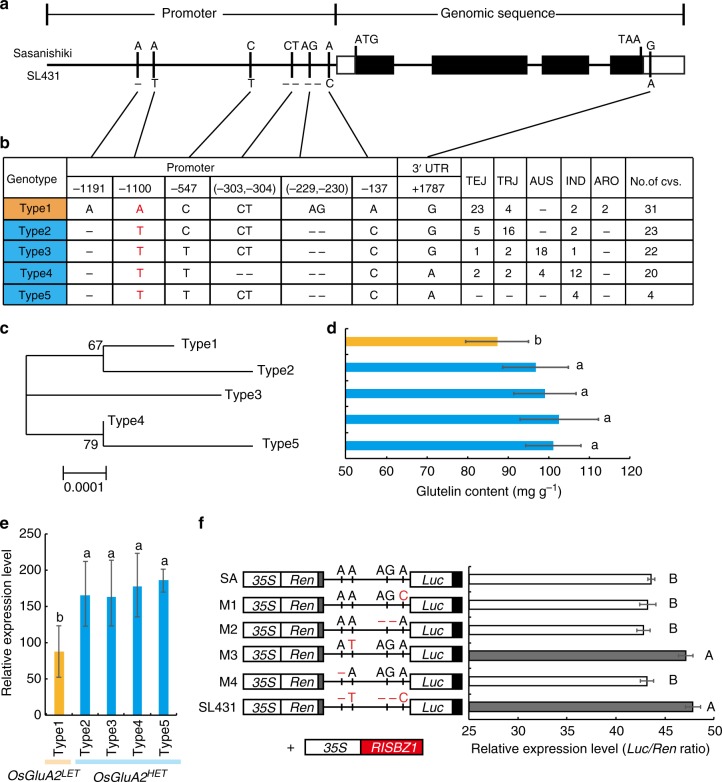
Haplotype analysis of *OsGluA2*. **a** Schematic of the gene structure and allelic variation on *OsGluA2* between Sasanishiki and SL431 indicated by vertical lines at bottom. **b** Haplotype analysis of the *OsGluA2* gene region from 100 rice cultivars. IND *indica* population, TEJ temperate *japonica* population, TRJ tropical *japonica* population, ARO aromatic population. **c** Phylogenetic tree of the five haplotypes. **d** Analysis of the glutelin content in representative varieties with five different haplotypes. *n* = 8 in type 1, *n* = 7 in type 2 and type 3, *n* = 6 in type 5, and *n* = 2 in type 5. **e** The relative expression level of the five haplotypes. Cultivar number of type 1, type 2, type 3, type 4, and type 5 haplotype was 8, 7, 7, 6, and 2, respectively. Three replicates of each cultivar were used for the analyses. **f** Transient expression assay of promoter activity in rice protoplasts. *n* = 10. Left, constructs with site-directed mutations at the four SNPs in the promoter region. Right, relative Luc/Ren values. Error bars, s.d. The presence of the same capital letter denotes a non-significant difference between them (*P* > 0.01). *P*-values were calculated by one-way ANOVA. Source data of Fig. 4d–f are provided as a Source Data file

To identify the natural variation of *OsGluA2*, we firstly sequenced the genomic region of *OsGluA2* from Sasanishiki and SL431. We found no nucleotide differences between the two cultivars in the coding regions of *OsGluA2*. Instead, six polymorphisms in the promoter region (2 kb) and one polymorphism in 3′-untranslated region (3′ UTR) were revealed (Fig. 4a).

In order to trace the distribution pattern of *OsGluA2*, we then sequenced the full-length *OsGluA2* genes of 100 accessions from different countries, including 31 temperate *japonica*, 24 tropical *japonica*, 22 *aus*, 21 *indica,* and 2 aromatic accessions (Fig. 4b). Phylogenetic analysis showed that *OsGluA2* diverges into five types (haplotypes) (Fig. 4c). To test the effect of these seven mutations on gene expression, we measured the transcript abundances of *OsGluA2* in these five types in endosperms at 15 DAF and their corresponding glutelin contents in mature seeds. The result indicates that the cultivars possessing type 1 tend to show significant lower expression levels and lower glutelin content than other types (Fig. 4d, e). These results suggest that four nucleotide mutations (SNP-1191, SNP-1100, InDel-229 to −230, and SNP-137) in the promoter region seems to be associated with the GPC diversity. Further, we conducted transient expression assays of the site-directed mutated promoter fragments of *OsGluA2* in rice protoplasts to test the effects of the four SNPs in the promoter region. Compared with the activity of the Sasanishiki promoter, the relative activity of the promoter fragment with one mutation (M3, −1100 position) was greatly increased (*P* < 0.01, one-way ANOVA), similar to SL431 promoter, whereas M1, M2, and M4 exhibited relatively low activities as same as Sasanishiki (Fig. 4f). Coincidentally, according to PLACE (plant *cis*-acting regulatory DNA elements) analysis, SNP-1100 was found to residue in a *cis*-regulatory element (BIHD1OS)^38^. These results indicate that the SNP-1100 might be functional SNP (FNP) accounting for the expression level differences between the *OsGluA2* alleles. Thus, this FNP was used to define the haplotypes of *OsGluA2* as two types, one is the low expression type (*OsGluA2*^*LET*^), while all others as the high expression type (*OsGluA2*^*HET*^).

### *OsGluA2* contributes to the divergence of *indica* and *japonica*

**Fig. 5:**
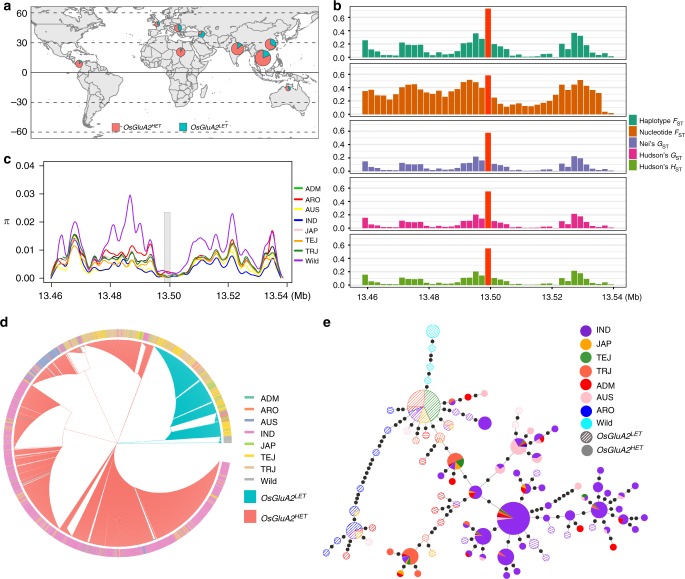
The geographic distribution, phylogenetic relationship and genetic difference of *OsGluA2*. **a** The geographic distributions among 3005 cultivated rice accessions. The types of *OsGluA2*^*LET*^ and *OsGluA2*^*HET*^ were indicated by green and red circles, respectively. **b** The parameters of genetic difference between *indica* and *japonica* ecotypes for *OsGluA2* and its flanking genomic regions. **c** Nucleotide diversity and selection analyses in *OsGluA2* and flanking regions (~80 kb). Genomic sequences of 3005 cultivated and 27 wild accessions were acquired from RFGB (http://www.rmbreeding.cn/Snp3k) and OryzaGenome (http://viewer.shigen.info/oryzagenome/), respectively. ADM admixed, ARO aromatic, IND *indica,* JAP unclassified *japonica*, TEJ temperate *japonica,* TRJ tropical *japonica*. **d** Phylogenetic relationship of *OsGluA2* generated from 3005 cultivated and 27 wild rice accessions. **e** Haplotype network of *OsGluA2*. Circle size is proportional to the number of samples for a given haplotype. Black spots on the lines indicate mutational steps between haplotypes

In order to investigate the natural variation of *OsGluA2* in rice germplasm, we further analyzed the genomic sequences of this gene in 3005 cultivated accessions, constitutive of cultivars from seven groups^37,39^. We noticed that the distribution of two functional types of *OsGluA2* is unbalanced in seven cultivated rice groups (Supplementary Table 8, *χ*^2^ = 1025.774, *P* < 0.0001). The cultivars of temperate *japonica* possessed the highest proportion of *OsGluA2*^*LET*^ (213 out of 317, 67.19%), while those of *indica* have the lowest level (70 out of 1755, 3.99%). In addition, the proportions of *OsGluA2*^*LET*^ in unclassified *japonica* (74 out of 131, 56.49%) and tropical *japonica* (141 out of 387, 36.43%) are also much higher than that of *indica*. These results indicate that *japonica* cultivars generally have higher proportion of *OsGluA2*^*LET*^ than *indica* cultivars. Moreover, we noticed that the proportion of *OsGluA2*^*LET*^ has the trend of increasing along with the increase of latitude, suggesting that a regional differentiation in *OsGluA2* (Fig. 5a), especially for *japonica*. We further investigated the parameters of genetic divergence for *OsGluA2* and its flanking regions between *indica* and *japonica* subspecies, including the estimates of haplotype and nucleotide *F*_*ST*_, Nei’s *G*_*ST*_, and Hudson’s *G*_*ST*_ and *H*_*ST*_ (Fig. 5b). All the estimates of these five parameters in *OsGluA2* locus are much higher than its flanking genomic regions. These results suggest there is genetic differentiation in *OsGluA2* between the two subspecies, which might contribute to the divergence of *indica* and *japonica*.

### *OsGluA2*^*LET*^ is derived from wild rice

In order to reveal whether artificial selection has contribution to the domestication of *OsGluA2*, the genomic information of 3005 cultivated rice and 27 *O. rufipogon* accessions were used to analyze the genetic diversity of this gene and its flanking regions (Fig. 5c and Table 2). The nucleotide diversity value (*π*) for this gene is much lower than its flanking regions in both cultivated and wild populations (*O. rufipogon*), suggesting that low nucleotide diversity in the locus of *OsGluA2* might be the result of natural selection rather than artificial selection. Moreover, all the estimation values of Tajima’s *D* for cultivated and wild populations are negative, indicating low-frequency polymorphisms in these regions. However, none of these values are statistically significant except for the wild population, suggesting that the locus of *OsGluA2* did not escape from neutral evolution during domestication.

**Table 2:** The estimated parameters of nucleotide diversity and Tajima’s *D* of *OsGluA2* and its flanking regions

	Parameter	ADM	AUS	ARO	IND	TEJ	TRJ	JAP	Cultivated	Wild
Upstream 40 kb	*π*	0.0070	0.0043	0.0076	0.0044	0.0051	0.0069	0.0067	0.0063	0.0125
	*θ*	0.0065	0.0066	0.0069	0.0083	0.0053	0.0065	0.0063	0.0095	0.0194
	Tajima’s *D*	0.2342	−1.0996	0.3464	−1.3539	−0.1449	0.1775	0.2363	−0.9493	−1.4035
*OSGluA2*	*π*	0.0008	0.0005	0.0010	0.0004	0.0005	0.0006	0.0005	0.0008	0.0017
	*θ*	0.0011	0.0011	0.0013	0.0012	0.0007	0.0006	0.0008	0.0019	0.0034
	Tajima’s *D*	−0.6403	−1.3330	−0.8070	−1.5523	−0.7923	0.1635	−0.8324	−1.5007	−1.9441*
Downstream 40 kb	*π*	0.0060	0.0046	0.0062	0.0035	0.0053	0.0060	0.0059	0.0053	0.0095
	*θ*	0.0055	0.0054	0.0055	0.0072	0.0048	0.0055	0.0047	0.0083	0.0150
	Tajima’s *D*	0.3125	−0.5139	0.4022	−1.4720	0.2731	0.2926	0.8691	−1.0362	−1.4514

Single asterisk (*) indicates significance level at *P* < 0.05 tested by computing 1000 coalescent simulations

The phylogenetic analysis of *OsGluA2* suggests that *OsGluA2*^*LET*^ was derived from *O. rufipogon* (Fig. 5d). In addition, a haplotype network was constructed to describe the evolutionary relationships and mutational steps using all 87 *OsGluA2* haplotypes (Fig. 5e). Among them, 39 and 48 haplotypes belong to the types *OsGluA2*^*LET*^ and *OsGluA2*^*HET*^, respectively. All the wild accessions contain the type *OsGluA2*^*LET*^, and the haplotypes mainly presenting in *japonica* accessions have the closest relationship with those in wild rice. These results imply that the *OsGluA2*^*LET*^ originated from *O. rufipogon*, as well as that at least one mutational event contributed to the origin of *OsGluA2*^*LET*^ in *japonica* accessions. The type *OsGluA2*^*HET*^, which is dominant in *indica* accessions, is a result of mutation from *OsGluA2*^*LET*^. In addition, we also noticed that 15 haplotypes (including 27 accessions) with the type *OsGluA2*^*LET*^ are the results of reverse mutation of *OsGluA2*^*HET*^.

## Discussion

Rice grain quality is a complex character, of concern to a wide range of consumers and breeders. GPC serves as a critical factor in determining rice nutrition quality, as well as eating and cooking quality. However, the genetic mechanism of GPC remains largely unclear yet, mainly due to the difficulty in mapping and cloning GPC related genes/QTLs. In the present study, we reveal that GPC variation in rice germplasm is mainly attributed to the glutelin content variation. Subsequently, two stably inherited QTLs, *qGPC-1* and *qGPC-10*, are identified in a cross of *indica* and *japonica*. *qGPC-10*, encoding the glutelin type-A2 precursor, is successfully cloned and functionally characterized. Our results signify a step toward unraveling the mechanism underlying natural variations of GPC between *indica* and *japonica* subspecies.

Rice is a widely distributed crop and there are many differences between *indica* and *japonica* subspecies. Like many other agronomic traits, GPC is also found to dramatically differentiate between the two subspecies, being generally higher in *indica* ones^14,15^. However, why is the GPC in *indica* accessions higher than in *japonica*? In the present study, we reveal that glutelin contents variation is the main reason causing GPC variation between *indica* and *japonica* germplasms, accounting for more than 70% (*R*^2^ = 0.739) of phenotypic variation (Fig. 1b, c). This result brings us some enlightenment that glutelin content should be regarded as a target for direct manipulation in rice quality improvement. Interestingly, in our study, *qGPC-10*, one of the environmentally stable QTL detected in the *indica* and *japonica* cross, is found to encode glutelin precursors and thus to regulate the glutelin synthesis and accumulation through the way of differentially transcriptional expression intensity. This stimulates us to explore that whether other glutelin synthesis-related genes are also responsible for the glutelin accumulation contribute to the GPC variation in the same way. To address this issue, 12 modern cultivars (six typical *indica* and six *japonica* cultivars) were employed to investigate the expression level of glutelin synthesis-related genes, it happens that there is a similar pattern, most of glutelin synthesis-related genes exhibit higher transcriptional expression levels in *indica* genetic background except for *GluB2* (Supplementary Figs. 5 and  6). Based on this phenomenon, it is reasonable to deduce that the expression level differences in glutelin synthesis gene may be the main cause of glutelin content variations between *indica* and *japonica* subspecies.

SSPs, accounting for ~90% of rice GPC, are storage nitrogen sources for the germinating seedlings and serve as a nutrient source for humans and livestock. *SSPs* genes constitute of multigene families. Previous studies showed that several mutations in a few genes have little effect on seed protein content and amino acid composition^40^. Kawakatsu et al. (2010) characterized glutelin, globulin, and prolamin knockdown lines in rice, and found that a reduction of one or a few SSPs could be compensated for by increasing in other SSPs at both the mRNA and protein levels. For example, Glu-less lines display marked reduction of GluA-2 and GluB-4, and bands corresponding to GluA-1, GluB-2, and GluB-1 are invisible on a stained gel, whereas levels of globulin 1 (Glb-1) and 13-kDa prolamins significantly increased. Similarly, they also found the 13-kDa prolamin-less transgenic seeds have high levels of glutelin and Glb-1 (ref. ^40^). However, in the present study, we find that the glutelin and prolamin contents alter in same directions, increase or decrease simultaneously in NILs, HpHc, and KO transgenic lines (Table 1). Moreover, this phenomenon is also observed in a similar case, in which reduced abundances of glutelin and prolamin are simultaneously observed in dry seeds of a globulin-deficient rice mutant, which is generated with RNA interference (RNAi)-induced suppression of globulin expression^41^. Thus, further studies are needed to address whether there is any common mechanism in corporately regulating the glutelin content and prolamin content in rice.

In the past decade, although numerous QTLs for GPC variation have been detected in rice germplasms^28–35^, few QTLs have been cloned except for *qPC1*/*OsAAP6* (ref. ^35^). However, the *OsAPP6* expression level is found to be associated with GPC variation only in *indica* accessions, and no correlation between *OsAAP6* expression level and GPC variation was detected in the *japonica* genetic background^35^. It seems that *OsAAP6* could be only used as a target gene to regulate GPC in *indica* breeding programs. Unlike *OsAAP6*, two haplotypes of *OsGluA2, OsGluA2*^*LET*^ and *OsGluA2*^*HET*^, are found to mainly residue in *japonica* and *indica* cultivars, respectively (Fig. 4b). The *OsGluA2*^*LET*^ exhibits a lower transcriptional level than *OsGluA2*^*HET*^, which corresponds with the glutelin content variation (Fig. 4c–e). So, our result suggests that the *OsGluA2*^*LET*^ allele from *japonica* accessions could be directly replaced with *OsGluA2*^*HET*^ allele to improve the nutrition quality in *japonica* cultivar development through marker-assisted selection. It should be noted that GPC is also found to be inversely correlated with palatability^4,32^. High GPC may lead to densely structured rice grains, which will result in hard and loose cooked rice, and thus poor palatability^12^. We previously identified a major QTL *qPC-1*. The introgression of the Habataki allele of *qPC-1* into a *japonica* background leads to a decrease in GPC, consequently leading to the decrease in nutritional quality of some content, whereas its palatability has been enhanced^28^. Therefore, in rice breeding practice, reducing rice GPC to some content has been becoming an important target for breeding good eating and cooking quality rice varieties. In fact, many famous commercial varieties, such as Koshihikari in Japan and Kongyu131 in north of China, their GPCs are usually less than 7%. In addition, some landrace or cultivars (LGC-1 mutant and W3660) with low glutelin contents (less than about 20% of total GPC) are identified and has been served as gene resource to develop new cultivars suitable for patients affected with diabetes and kidney failure^11^. Hence, the new strategy for balancing nutrition quality and eating and cooking quality in rice grain is urgent to develop, and more valuable gene resources are needed to explore. The cloned *OsGluA2* in the current study as well as *OsAAP6* could serve as the targets to manipulate through gene editing or molecular marker-aided selection in rice quality improvement.

## Methods

### Plant materials and cultivation

The CSSL population derived from a cross of Sasanishiki (*japonica*) and Habataki (*indica*) with 39 lines in total was employed for identification of QTLs controlling GPC, which was introduced from National Institute of Agrobiological Sciences, Japan (http://www.rgrc.dna.affrc.go.jp/stock.html). The CSSLs population and two parents were planted in three environments for GPC measurements, e.g. Yangzhou (Jiangsu Province, China, 32^°^24′N) in 2014 and 2015, and Lingshui (Hainan Province, China, 18^°^30′N) in 2014. For fine mapping of *qGPC-10*, the F_2_ and F_3_ populations were generated from a cross between SL425 and Sasanishiki, and cultivated in Yangzhou in summer seasons for GPC investigation in 2014 and 2015, respectively, with an interplant spacing of 10 × 25 cm for transplanting. Other materials used in this study are listed in Supplementary Data 1 and  2, including a core germplasm mainly from China (205 *indica* and 197 *japonica* cultivars) for GPC investigation^36,37^ and 100 accessions (31 temperate *japonica*, 24 tropical *japonica*, 22 *aus*, 21 *indica*, and 2 aromatic accessions) from worldwide for genetic diversity analysis on *OsGluA2* (ref. ^34^). The amount of fertilizer applied per hectare was 50 kg nitrogen, 60 kg phosphorus, and 90 kg potassium as the basal fertilizer, followed by 85 kg nitrogen at tilling stage and 25 kg at the booting stage.

### Map-based cloning

A total of 790 F_2_ plants with extreme phenotypes derived from the cross of SL431 and Sasanishiki were used for genetic analysis and mapping of *qGPC-10* on chromosome 10. An additional 1085 F_3_ individuals were then used for screening recombinants for fine mapping of *qGPC-10*. The genomic DNA fragments corresponding to the candidate gene in SL431 and Sasanishiki were sequenced and analyzed. A list of the markers used for QTL analysis and positional cloning is given in Supplementary Table 9.

### Trait measurement

Rice grains of each sample were harvested at ~40 DAF and air-dried, then stored at room temperature for 3 months for measuring grain quality and yield traits. For each plant, 15–20 g of brown rice were prepared for the measurement of grain quality, including AC, GC, starch content, and GPC. The GPC was carefully determined by near-infrared spectroscopy using Infratec 1241 Grain Analyzer (Foss Tecator, Sweden) equipped with STM model^42^. Briefly, approximately 15–20 g dry seeds per sample were dehulled into brown rice using a TR 200 dehuller (Kett, Tokyo, Japan) and loaded into the sample cup. Three scans per sample were used in data analysis. Data of seed protein contents were presented on a 0% moisture basis. These calibrations involved more than 500 rice samples that varied in GPC. Crude GPC and storage protein fractions in rice flour were also determined by the Kjeldahl method using a Kjeltec 2300 Autoanalyser (Foss AB, Sweden)^28^. A nitrogen conversion factor of 6.25 was used to calculate the GPC.

### Protein extraction from rice grains and the SDS-PAGE assay

Mature dry seeds of rice plants were harvested, dehulled and ground to a fine powder using a mortar and pestle at room temperature. The total seed proteins were extracted from the powder (100 mg) with 1 mL of SDS-urea buffer (4% SDS, 8 M urea, 0.25 M Tris-HCl (pH = 6.8), 20% glycerol, and 5% β-mercaptoethanol (β-ME)) by shaking overnight. The supernatant was removed after centrifugation at 1500 ×*g* at 4 °C for 20 min. The total seed protein (10 μL) was separated by gradient SDS-PAGE gels (15%)^10^. The densitometry of the gels was measured by using the ImageJ software.

### RNA extraction, cDNA preparation, and quantitative reverse transcriptase PCR

Total RNAs were extracted from rice various tissues using the Trizol reagent (Invitrogen). Total RNA was used for synthesizing first-strand cDNA with a reverse transcription kit (TOYOBO). Quantitative reverse transcriptase (qRT-PCR) was performed on an ABI 7500 instrument using SYBR Green PCR Mastermix followed the manufacturer’s instructions (Takara, R820). All assays were performed with at least three biological replicates. Rice *TUBLIN* gene serves as the internal control to normalize gene expression. A list of primers used for qRT-PCR is given in Supplementary Tables 9 and  10.

### Vector constructions and plant transformation

Various DNA fragments were amplified and inserted into different binary vectors for plant transformation. The information of relevant primer sequences was given in Supplementary Table 9. To prepare the complementation construct (HpHc), we first obtained ~2 kb promoter fragment and the whole-genomic sequence of *OsGluA2* by PCR amplification from SL431 and then inserted them into the plant binary vector pCAMBIA1301. To prepare the construct for CRISPR-Cas9 (KO-*OsGluA2*), a 20 bp fragment with NGG (PAM) at 3′ end from the second exon of *OsGluA2* was introduced into the vector pC1300*-*Cas9. The promoter fragments of *OsGluA2* were amplified from Sasanishiki and SL431, respectively, and then were inserted into the expression vector pCAMBIA1391Z-GUS, namely *qGPC-10*^*S*^::GUS and *qGPC-10*^*H*^::GUS. The complementation and GUS constructs were introduced into the rice cultivar Sasanishiki and the KO-*OsGluA2* construct was introduced into the CSSL line SL431. All the resulting constructs were introduced into *Agrobacterium tumefactions* strain EHA105 and transferred by *Agrobacterium*-mediated transformation into receipts.

### Analysis of *GUS* gene expression

For histochemical analysis, rootlets, leaves, leaf sheaths, and stalks cut into 5 mm sections and maturing seeds at 5, 10, 15, 20, and 25 DAF sectioned longitudinally with a razor blade were subject to incubation in a solution with 10% methanol, 0.5% Triton X-100, 50 mM NaPO_4_, and 1 mM X-gluc (5-bromo-4-chloro-3-indolyl-β-d-glucuronic acid), at 37 °C for 12 h under the dark condition. After GUS staining, chlorophyll was removed using 75% ethanol before taking pictures. Fluorometric assays of GUS activities were conducted according to Kawakatsu et al.^9^, with maturing seeds at 15, 20, and 25 DAF used as samples. For each construct, ten independent transformants were subjected to fluorometric GUS assays.

### Transmission electron microscopy analyses

Transverse sections (1 mm thick) of immature 15 DAF seeds from wild-type and transformants were fixed for over 12 h in 0.2 M phosphate buffer (pH 7.3) containing 2.5% glutaraldehyde at 4 °C. The sections were post fixed on ice for 1 h in 1% osmium tetroxide in 0.1 M phosphate buffer, dehydrated in a graded ethanol series, and embedded in Epon 812. The specimens were sliced into ultrathin sections by using an ultramicrotome (Leica EM, Austria) with a diamond knife (Diatome, Switzerland), and stained with a solution of uranyl acetate and lead citrate. Microscopic observation was performed using a transmission electron microscope (CM100, Holland) at an accelerating voltage of 100 kV. The area of PBs in each sample was determined using ImageJ software (NIH).

### Amino acid analysis

For total amino acid analysis, 10 mg of rice power of each sample was hydrolyzed with 1 mL of 6 N HCl (Sigma, USA) in a 2 mL screw-cap tube before adding 10 nmol l-(+)-norleucine (Wako Pure Chemicals, Japan). The samples were then heated at 110 °C for 24 h, followed by the treatment of 6 h at 65 °C in order to evaporate HCl completely. The residue was then dissolved in 1 mL Na-S™ buffer and centrifuged at 1600 × *g* for 10 min at room temperature. The supernatant was filtered with a 0.45 μm nylon membrane syringe filter (Pall Life Sciences, USA) and transferred to an autosampler bottle for amino acid analysis. HPLC data were normalized with the level of l-(+)-norleucine per sample. Three biological replicates were designed for each sample. Seventeen amino acids were measured, including alanine (Ala), arginine (Arg), aspartic acid (Asp), cysteine (Cys), glutamic acid + glutamine (Glu), glycine (Gly), histidine (His), isoleucine (Ile), leucine (Leu), lysine (Lys), methionine (Met), phenylalanine (Phe), proline (Pro), serine (Ser), threonine (Thr), tyrosine (Tyr), and valine (Val).

### Transient expression assays in rice protoplasts

A series of mutated promoters of *OsGluA2* were cloned into the pGreenII 0800-LUC vector^43^, and a Renilla luciferase gene was used as an internal transformation control to provide an estimate of the extent of transient expression in the same construct. Previous studies showed that the RISBZ1 (rice seed b-Zipper 1) regulatory factor expresses with rice *SSP* genes coordinately, and activates transcription from *SSP* gene promoters in transient expression assays^44–46^. The full-length cDNAs of *RISBZ1* was fused into pCambia1300-221-Flag vector. The different combinations of vectors were co-transfected into rice protoplasts^47^ by PEG-mediated transformation. Ratios of *LUC* to *Ren* activity were calculated to define relative promoter activity. Ten biological replicates were designed. Relevant PCR primer sequences were provided in Supplementary Table 9.

### Nucleotide diversity and evolutionary analyses

The genomic sequences of 3005 cultivated and 27 wild accessions were obtained from Rice Functional Genomics and Breeding Database^37,39^ (RFGB, http://www.rmbreeding.cn/Snp3k) and OryzaGenome^48^ (http://viewer.shigen.info/oryzagenome/), respectively. The distribution of two functional types of *OsGluA2* in different cultivated rice groups was compared using chi-square (*χ*^*2*^) test. The geographical information of cultivated rice groups was acquired from RFGB, and was marked on map using R software to observe geographic distribution characteristics of two types. The parameters of genetic difference between *indica* and *japonica*, including haplotype and nucleotide *F*_ST_, Nei’s *G*_ST_, and Hudson’s *G*_ST_ and *H*_ST_ were calculated within per 2 kb regions using *PopGenome* package in R software^49^. The estimates of nucleotide diversity (*π* and *θ*) and Tajima’s *D* for each rice group in Os*GluA2* and flanking regions (~80 kb) were calculated using DnaSP V5 (ref. ^50^). In addition, nucleotide diversity curves were acquired using 2000 bp window and 100 bp step length. In order to evaluate the evolution of *OsGluA2*, a phylogenetic tree and haplotype network were constructed using all the polymorphic sites with minor allele frequency ≥0.005 in this locus. The phylogenetic tree for Os*GluA2* was constructed using UPGMA method by Mega7.0 (ref. ^51^), while the haplotype network was built by *pegas* package^52^.

### Reporting summary

Further information on research design is available in the Nature Research Reporting Summary linked to this article.

## Supplementary information

Supplementary Information

Peer Review File

Description of Additional Supplementary Files

Supplementary Data 1

Supplementary Data 2

Reporting Summary

Source Data

## Data Availability

Data supporting the findings of this study are available within the paper and its Supplementary Information files. A reporting summary for this article is available as a Supplementary Information files. The datasets generated and analyzed during the current study and plant materials are available from the corresponding author upon reasonable request. The source data underlying Figs. 2a–c, 2e–h, 3a, 3c, 3d, 3f, and 4d–f, Table 1, Supplementary Figures 1b, 1d, 2c, 3c, 4, 5, and 6, as well as Supplementary Table 7 are provided as a Source Data file.
