# Supplementary material for: Natural variation of OsGluA2 is involved in grain protein content regulation in rice
Source: Nat Commun. 2019 Apr 26;10:1949. doi: 10.1038/s41467-019-09919-y (PMC6486610; doi:10.1038/s41467-019-09919-y)
Supplement: Supplementary file 1 — Supplementary Information [file 41467_2019_9919_MOESM1_ESM.pdf]

Natural variation of *OsGluA2* is involved in grain protein content regulation in rice

Yang *et al.*

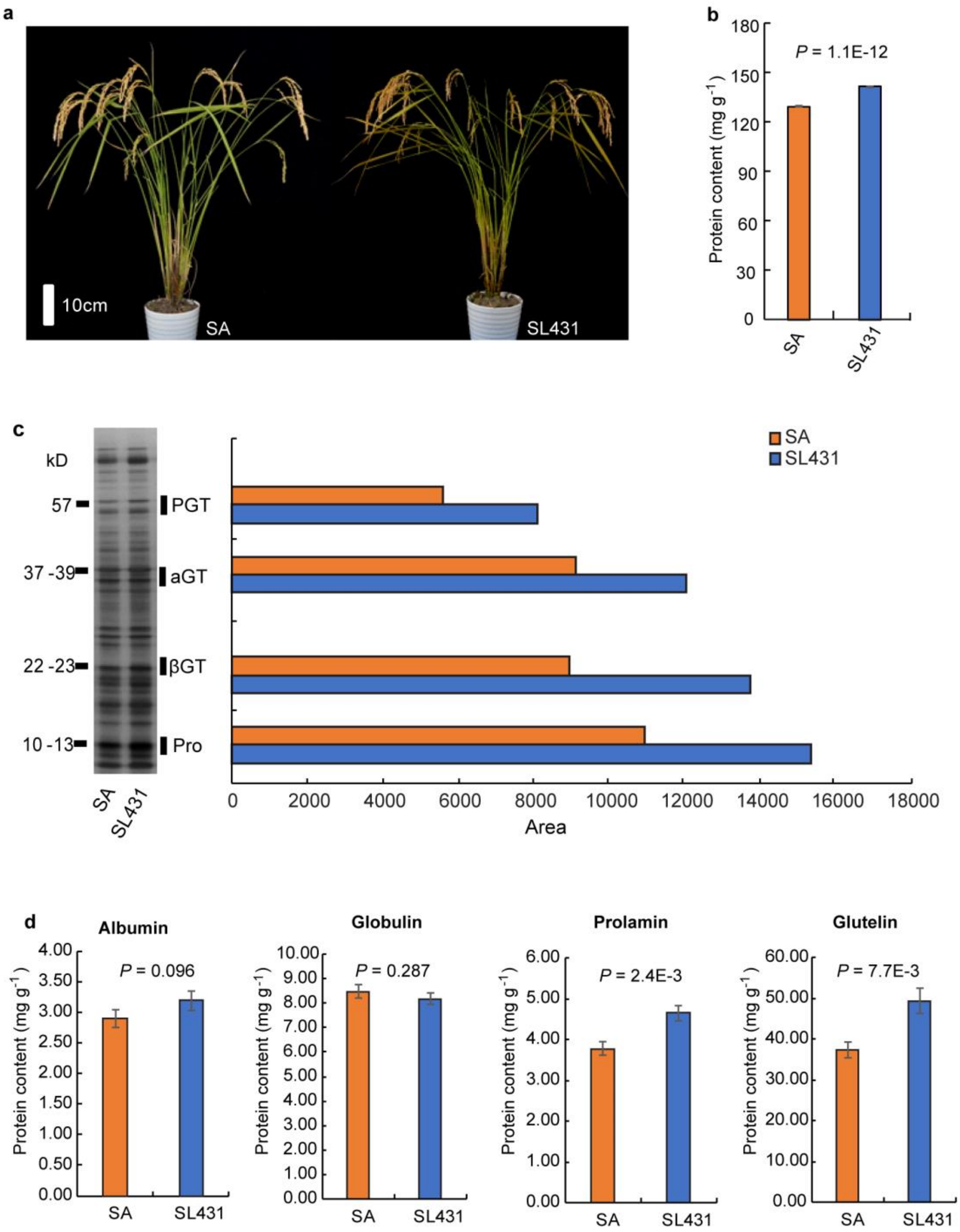

Supplementary Figure 1. Morphologies and protein contents comparison between SL431 and Sasanishiki. **(a)** Gross morphologies of Sasanishiki and SL431. Scale bar = 10 cm. (**b**) Total protein content of Sasanishiki and SL431. (**c**) SDS-PAGE for storage protein profiles of dry seeds of Sasanishiki and SL431 and quantitative analysis by ImageJ software. pGT, proglutelins; aGT, glutelin acidic subunits; βGT, glutelin basic subunits; Pro, prolamin. (**d**) Comparison of grain protein fraction contents between Sasanishiki and SL431. Error bars, s.d., n = 3. *P*-values were produced by independent-samples *t*-test. Source data of Supplementary Figure 1b and 1d are provided as a Source Data file.

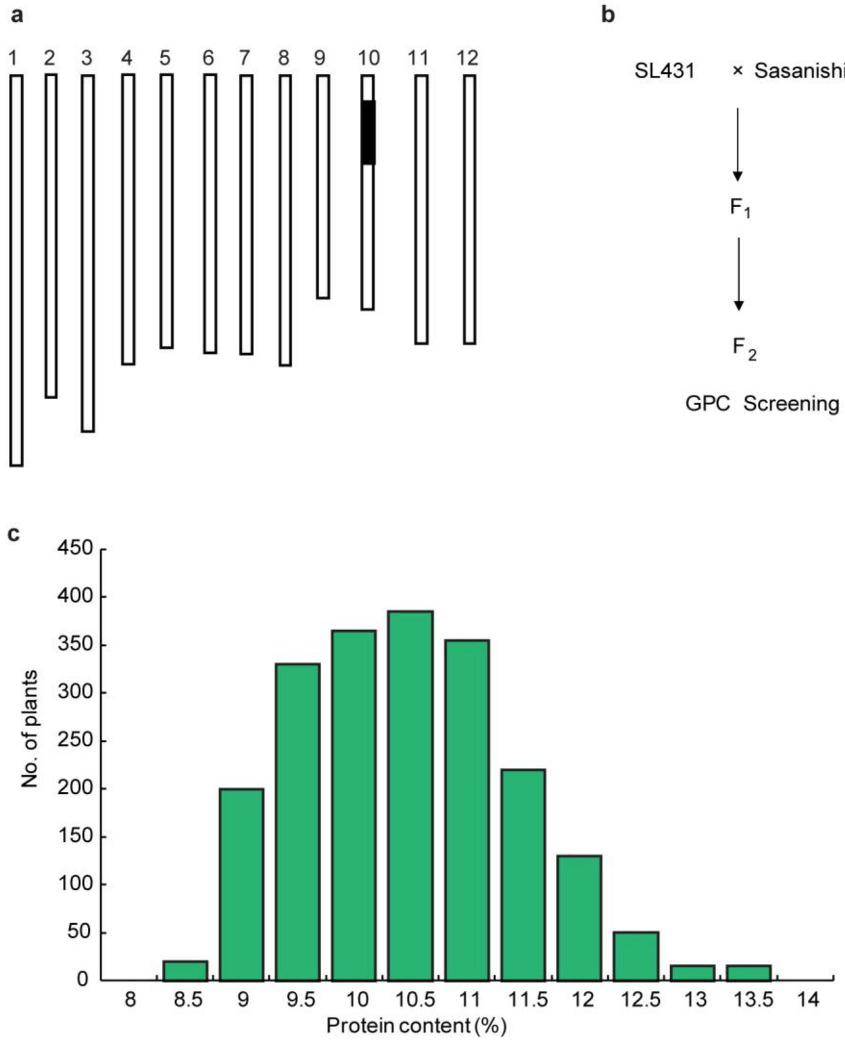

Supplementary Figure 2. SL431 harboring *qGPC-10* was used to develop mapping population. **(a)** Graphical genotype of SL431. White bar, genomic region from Sasanishiki; black bar, genomic region from Habataki. **(b)** Schematic to generate F_2 population from SL431 × Sasanishiki. (**c**) Frequency distribution of the secondary F_2 population from the cross of SL431 and Sasanishiki. Source data of Supplementary Figure 2c are provided as a Source Data file.

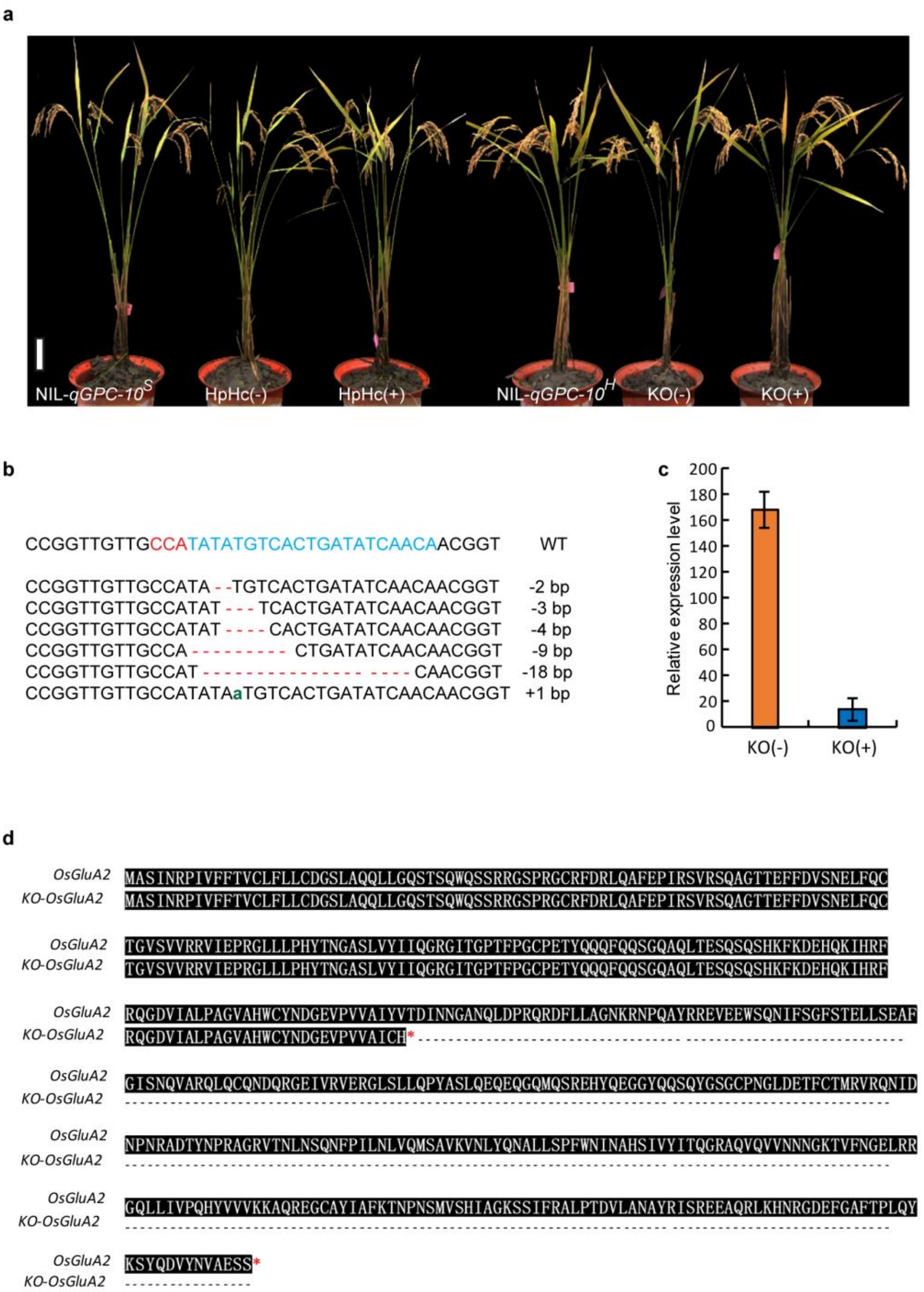

Supplementary Figure 3. The genetic information of transgenic plants. (**a**) Comparison of plant architectures of NILs, transgene-positive plant (+) and negative counterparts (-) in T_2. (**b**) Parts of mutations at the *GluA2* locus in T_0 generation plants generated by CRISPR-Cas9. The targeted sequence is highlighted in blue and the PAM sequence in red. Mutations with 1 bp insertions are in green lowercase letters. The deleted sequences are shown by red hyphens. (**c**) Real-time PCR to show that the expression of *OsGluA2* was down-regulated in transgenic rice lines at 15 DAF. Error bars, s.d., n = 3. (**d**) Polypeptide sequences of *OsGluA2* from the wild-type and KO-*OsGluA2* mutant generated using CRISPR-Cas9 technology, showing a TA deletion in *OsGluA2* from KO-*OsGluA2* mutant, leading to truncation of the *OsGluA2* protein. *, stop codon. Source data of Supplementary Figure 3c are provided as a Source Data file.

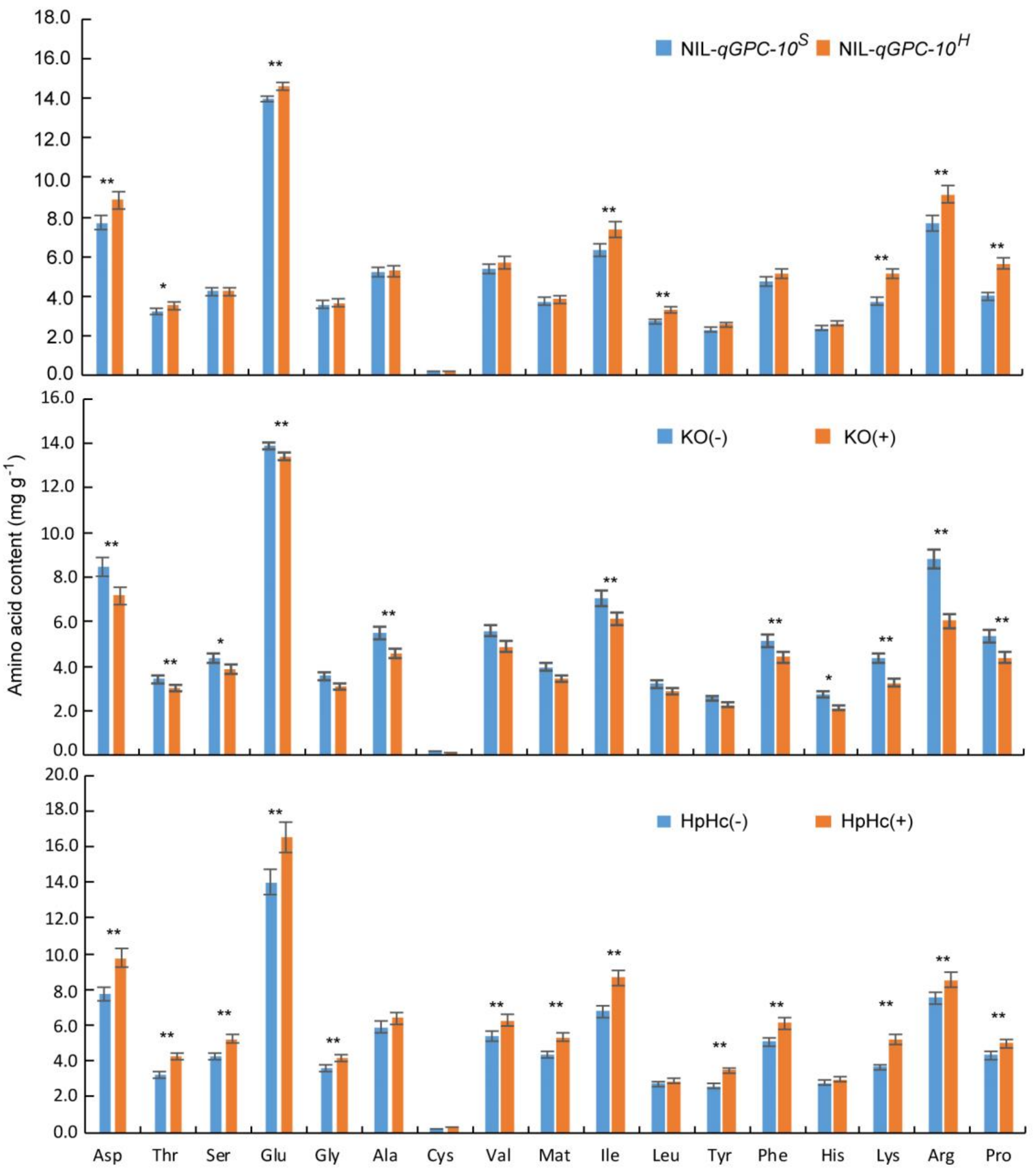

Supplementary Figure 4. Amino acids contents of the grain in NILs, KO and HpHc. (+) and (–) indicate transgene-positive and negative T_3 plants, respectively. Error bars, s.d., n = 3, significant differences at $*P < 0.05$ and $**P < 0.01$. Significant differences are based on independent-samples *t*-test. Source data are provided as a Source Data file.

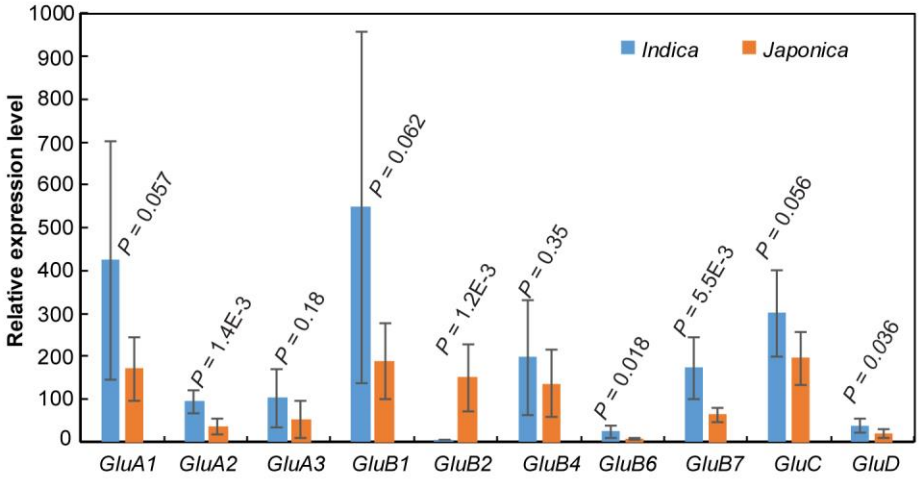

Supplementary Figure 5. Comparison of expression levels of glutelin gene family between the six *indica* and six *japonica* cultivars. Six *indica* cultivars: Yuexiangzhan, Nanjing 11, Miyang 46, Guichao 2, Newrex, and 9311; six *japonica* cultivars: Huajing 3, Nanjing 46, Guanglingxiangjing, Taibei167, Yanjing 5, and Chunjing 06**.** Error bars, s.d. Data represent gene expression average of six cultivars in each subspecies. For each cultivar, samples from three biological replicates were collected for analysis. *P*-values were produced by independent-samples *t*-test. Source data are provided as a Source Data file.

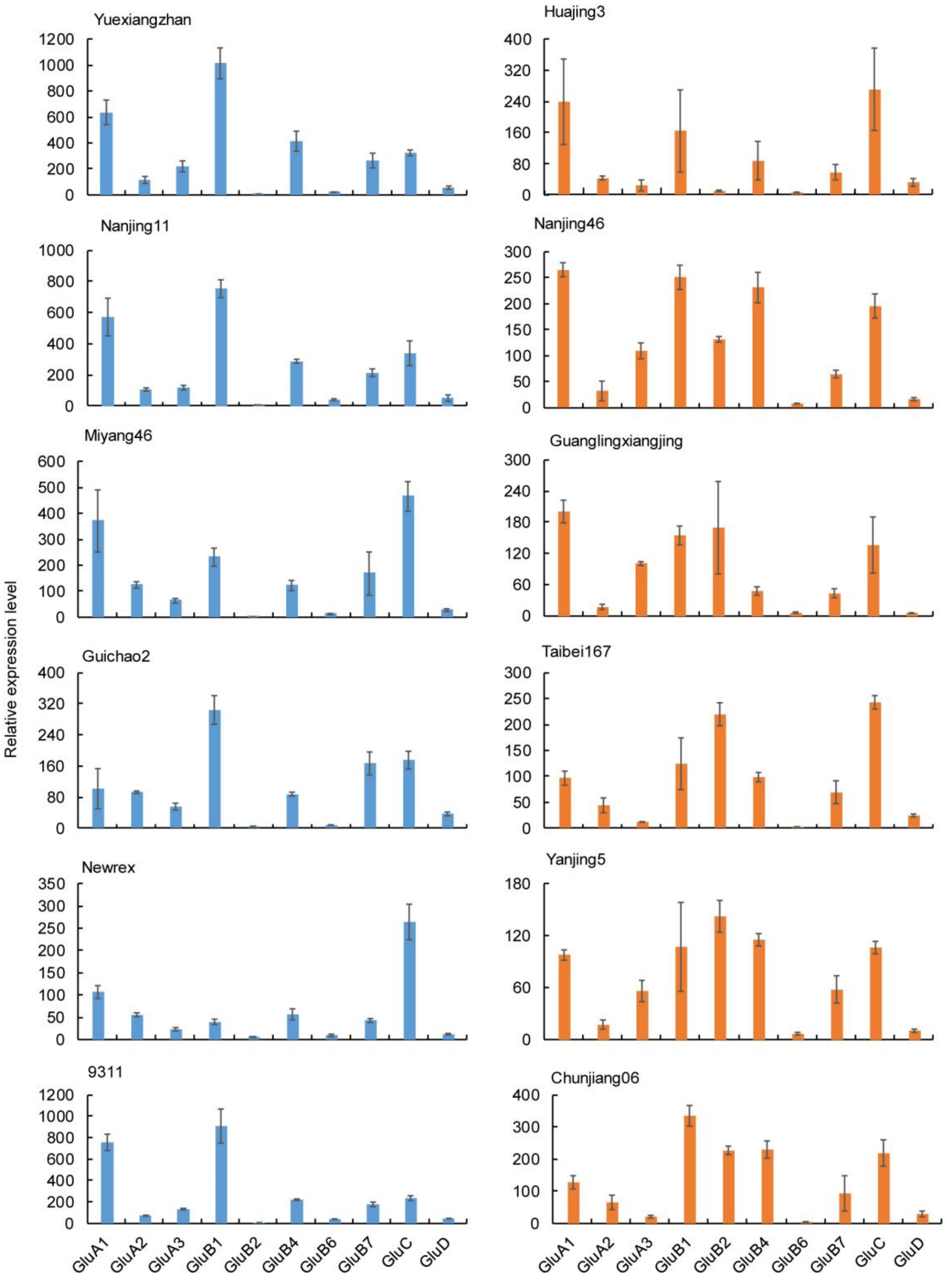

Supplementary Figure 6. The detail information of expression patterns of glutelin synthesis gene family in the 12 typical *indica* and *japonica* cultivars. Six *indica* cultivars: Yuexiangzhan, Nanjing 11, Miyang 46, Guichao 2, Newrex, and 9311; six *japonica* cultivars: Huajing 3, Nanjing 46, Guanglingxiangjing, Taibei167, Yanjing 5, and Chunjing 06. Same datasets as Supplementary Figure 5 were used to plot the bar charts. Error bars, s.d., n = 3. Source data are provided as a Source Data file.

Supplementary Table1. The distribution of GPC among 402 accessions in 2012 and 2013.

Types	No. of accessions	Year	GPC range (%)	Means ± SEM (%)	P^a
Japonica	197	2012	5.33 – 11.17	7.99 ± 0.07	2.59×10^{-52}
		2013	6.85 - 13.93	9.91 ± 0.08	
Indica	205	2012	6.70 - 13.30	9.29 ± 0.09	3.56×10^{-46}
		2013	8.54 - 14.83	11.19 ± 0.08	
Total	402	2012	5.33 - 13.93	8.66 ± 0.06	2.98×10^{-77}
		2013	6.85 - 14.83	10.56 ± 0.07	

[a]*P*-values were based on independent-samples *t*-test.

Supplementary Table2. The distribution of GPC with different types among 402 accessions in 2013.

Types	Total	No. of samples		
		High[a]	Medium[b]	Low[c]
Japonica	197	19	141	36
Indica	205	31	152	22
Total	402	50	293	58

[a]GPC $\geq \bar{x} + sd$; [b]$\bar{x} - sd <$ GPC $< \bar{x} + sd$; [c]GPC $\leq \bar{x} - sd$.

Supplementary Table3. GPC of CSSLs in 2014 and 2015.

Environments	Means (%)		GPC range (%)
	Sasanishiki	Habataki	
2014 Yangzhou	8.8	12.1	8.6 - 11.0
2014 Hainan	8.4	9.4	7.8 - 9.4
2015 Yangzhou	8.7	11.7	7.7 - 11.2

Supplementary Table 4. Genetic effects of QTLs for GPC in three environments using a CSSL population derived from a cross between Habataki and Sasanishiki.

QTL	Marker	LOD	Additive effect[a]	PVE (%)[b]
2014 Yangzhou				
qGPC-1	RM7124	2.798	-0.257	11.803
qGPC-6	RM3408	2.535	0.277	10.521
qGPC-9-1	RM3808	3.397	0.397	14.882
qGPC-10	RM7217	3.933	0.435	17.824
qGPC-12	RM6998	3.452	0.559	15.169
2014 Hainan				
qGPC-1	RM7124	6.065	-0.560	14.439
qGPC-3	RM3525	2.917	0.2516	5.672
qGPC-8	RM6369	4.980	0.489	11.047
qGPC-9-2	RM6971	8.028	0.359	21.809
qGPC-10	RM7217	4.106	0.310	8.605
qGPC-10-2	RM1859	2.494	0.235	4.945
qGPC-11	RM5824	3.688	0.289	7.533
qGPC-12	RM6998	5.751	0.540	13.413
2015 Yangzhou				
qGPC-1	RM7124	4.112	- 0.609	16.598
qGPC-3	RM3525	4.042	0.358	16.246
qGPC-8	RM6369	3.136	0.516	11.902
qGPC-9-3	RM6797	3.804	0.416	15.061
qGPC-10	RM7217	2.884	0.490	10.776

[a] Positive and negative values of additive effects indicate the alleles from Habataki and Sasanishiki with increasing effects, respectively.

[b] Percentage of total phenotypic variance explained by the QTL.

Supplementary Table 5. Grain protein contents of F_3 recombinants.

NO.	Protein content of F_3 recombinants							
	**R1**	**R2**	**R3**	**R4**	**R5**	**R6**	**R7**	**R8**
1	9.8	10.6	10.5	10	9.8	10	10.9	10.9
2	9.5	10.8	10.7	9.6	9.9	10.5	10.8	10.8
3	9.7	10.9	10.3	9.9	9.8	10.5	10.6	10.3
4	9.9	10.4	10.6	10		10.7	10.5	10.5
5	9.5	11.2	10.7	10		10.5	10.4	11.2
6	9.6	11	10.2	9.7		10.4	10.6	11
7	9.8	10.7	10.5			10.4	10.8	10.7
8	9.3	10.5	10.3				10.8	10.9
9	9.5	10.3	10.5				10.8	11
10	10.0	10.6	10.8				10.9	10.9
11	9.9	10.4	10.5					10.8
12	10.2	11.4	10.6					
13	10.1	10.7	10.7					
14	9.9	10.9	10.4					
15	10.2	10.6	10.1					
16	10.1	10.7	10.6					
17	9.9	10.7	10.6					
18	10.2	10.5	10.8					
19	10.1	11	10.4					
20	9.6	10.5	10.6					
Mean ± sd	9.8 ± 0.27	10.7 ± 0.28	10.5 ± 0.19	10.0 ± 0.17	9.8 ± 0.05	10.5 ± 0.11	10.7 ± 0.17	10.8 ± 0.24
Sig.[a]	c	a	b	c	c	b	a	a

[a] Significant difference are based on one-way ANOVA. The presence of the same lowercase letter denotes a non-significant difference between them ($P > 0.05$).

Supplementary Table 6. The four candidate genes in the 35 Kb long region.

Locus Identifier	Putative Function	Position
LOC_Os10g26050	methionyl-tRNA syntheses, putative, expressed	13492784 - 13496772
LOC_Os10g26060	glutelin, putative, expressed	13497239 - 13499435
LOC_Os10g26070	pentatricopeptide, putative, expressed	13501851 - 13503816
LOC_Os10g26110	decarboxylase, putative, expressed	13524139 - 13525925

Supplementary Table 7. Yield component traits of near-isogenic lines and transgenic plants.

Trait	1000 - grain weight (g)	Panicle length (cm)	Number of panicles per plant	Number of grains per panicle	Plant height (cm)
NIL-*qGPC-10*^{S}	20.36 ± 0.33	20.2 ± 0.3	11.8 ± 1.3	118.2 ± 3.6	83.3 ± 0.7
NIL-*qGPC-10*^{H}	20.49 ± 0.22	19.9 ± 0.4	11.2 ± 0.8	116.8 ± 2.4	83.2 ± 0.6
P^a	0.52	0.29	0.41	0.55	0.96
HpHc (-)	20.34 ± 0.16	19.7 ± 0.6	11.8 ± 0.8	117.6 ± 4.4	83.7 ± 0.4
HpHc (+)	20.54 ± 0.23	20.1 ± 0.4	11.6 ± 0.5	114.4 ± 2.1	83.6 ± 0.4
P^a	0.15	0.23	0.66	0.18	0.64
KO(-)	20.57 ± 0.18	19.6 ± 0.3	10.8 ± 1.2	112.4 ± 5.7	83.4 ± 0.3
KO(+)	20.49 ± 0.13	19.8 ± 0.5	11.4 ± 0.8	114.2 ± 4.6	83.0 ± 0.6
P^a	0.48	0.39	0.42	0.60	0.18

[a]*P*-value were produced by independent-samples *t*-test. All data are given as means ± s.d., n = 5. Source data are provided as a Source Data file.

Supplementary Table 8. Distribution of two functional types of *OsGluA2* in 3,005 cultivated rice accessions.

Ecotypes	*OsGluA2*^{HET}	*OsGluA2*^{LET}
ADM	91	41
ARO	23	45
AUS	201	14
IND	1685	70
JAP	57	74
TEJ	104	213
TRJ	246	141

ADM, admixed; ARO, aromatic Basmati/sadri; AUS, aus/boro; IND, *indica*; JAP, *japonica*; TEJ, temperate *japonica*, TRJ, tropical *japonica*

Supplementary Table 9. The primers for map-based cloning and functional analysis of *OsGluA2*

Usage	Name	Type	Forward primer (5' - 3')	Reverse primer (5' - 3')
Fine mapping	YYH-1	InDel	catcgtatcgggtgactta	cgagggacctaaagcaaa
	YYH-4	InDel	aaggattggatgtgggaagc	agcaacttcgggatgggt
	Y1	CAPs	gcccaacacttccagatt	agcgatggctatggtatg
	Y3	CAPs	ccactcactaaatacgctaa	agtgacctttgcccagt
	Y5	InDel	aattcgtctcgcagtttacagg	ggctgtccacacagataagtacg
	Y6	InDel	tcggtctctgtgccgtgattagg	caccagcgcagcaactaacatcc
Sequencing	Sq-1	InDel	caccttcctcgtatccag	cactcctttcaccgttct
	Sq-2	InDel	gaacaccgtctttccatt	gcattatcaagaaggagga
	Sq-3	InDel	gctgttgagatttgtaaccctt	ttgtgcgatggctcccta
	Sq-4	InDel	taactcggcggacaacag	tgaaccagcactcaagaa
	Sq-5	InDel	ccaacaccctactgctaa	aagtcttccctgtttacg
	Sq-6	InDel	aaaccaacccaagcaacc	cctttactcaaccctcacg
qRT-PCR	RT-qGPC-10		tgcttgttcctcttgtgcga	tatggcaacaaccggcactt
	RT-26050		gtcgtcgagagagagtcaaac	cgatgatgttccccaggtg
	RT-26070		attagaaatggggatgtgggtt	gctttctcaatgtttccacact
	RT-26110		gacttcctcgccggctacta	gaagtaggcgaagaagctgg
	Real-tublin		gctccgtggcggtatcat	cggcagttgacagccctag
Vector construction	HpHc		aaaactgcaggttgactgccctgaactctga	acgcgtcgactccatttctcctccgttgcatttaa
	GUS-H		cgcggatcctgtaggactaatgaactgaatgctt	cccaagctttgtatgctctttactcaaccctcac
	GUS-S		cgcggatcctgtaggactaatgaactgaatgctt	cccaagctttgtatgctctttactcaaccctcac
	KO-PAM		ggcagttgatatcagtgacatata	aaactatatgtcactgatatcaac
	PRO		ccccctcgaggtcgaccccccttctacattttgttctgg	ttggcgtcttccatgggttgttgtaggactaatgaactg
	M1		tgtaccacacttcatatatcctgagtcacttcatgtctggacat	atgtccagacatgaagtgactcaggatatatgaagtgtggtaca
	M2		atccatgtcatattgcaaaagaaagagaaagaacaacacaatgc	agcattgtgttgttctttctctttcttttgcaatatgacatggat
	M3		taattaatcatgcgctaatatgtcactctgtttttcgtact	agtacgaaaaacagagtgacatattagcgcatgattaatta
	M4		ctctgtgtttttgccaaaaaaaaatgtataggaaagttgcttttaa	ttaaaagcaactttcctatacattttttttggcaaaaaacacagag
	RISBIZ1		aggatccccgggtaccatggagcacgtgttcgccgtcgac	ggggaaattcgagctcctactgaagctccatgttgacaag

Supplementary Table 10. Primer sets used for qRT-PCR of the 32 genes involved in metabolisms of storage starch and storage protein and energy.

Name	FL-cDNA	Annotated name	Forward primer (5'-3')	Reverse primer (5'-3')	Reference
10kD Prolamin	AK108254	10 kD prolamin	TGCAGTATTTCCCACCAACA	ACATGAACATGGCTGTGGAG	She et al (2010)[1]
13kD Prolamin	AK242306	13 kD prolamin	CACAGCGCAGTTTGATGTTT	GCTTGCCGCAATGCTATACT	She et al (2010)[1]
17kD Prolamin	AK242322	17 kD prolamin	TTTGATGCTTGCACCTATGG	GCAGCTGCTCAGTTTTAGCC	She et al (2010)[1]
19kD Globulin	AK287940	19 kD globulin precursor	GCCAGTAATTGCAGGGGATA	AGGTCACCACCAACGTAAGC	She et al (2010)[1]
GluA1	AK242245	Glutelin A1	CATTTGAGCCAATTCGGAGT	GGCCTGATTGTTGGAACTGT	She et al (2010)[1]
GluA2	AK107314	Glutelin A2	TGCTTGTTCCTCTTGTGCGA	TATGGCAACAACCGGCACTT	In this study
GluA3	AK107217	Glutelin A3	TGAAAACCAACCCTGACTCC	ACTCATCTCCCCTGTTGTGC	She et al (2010)[1]
GluB1	AK107343	Glutelin B1	GCCAAAGTCAGAGCCAAAAG	GAACCAATGTGCAACACCAG	She et al (2010)[1]
GluB4	AK242872	Glutelin B4	GCGACCAGAAGGCTACAAAG	TTGCTTGTTGATCGTTGCTC	She et al (2010)[1]
GluC	AK064478	Glutelin C	CACAAGGGCCAATAGCCAGA	GGTCACGTACATCACCGTGT	Kim et al. (2013)[2]
GluD	AY429650	Glutelin D	AAGACAGAGCGACCAAGCTC	ATGTGCAACACTAGCCGGAA	Kim et al. (2013)[2]
RAG2	AK107328	Seed allergen RAG 2	AGGTAGTGTTCTCGGCGTTG	GTACATCGGGTAGCCCATTC	She et al (2010)[1]
PDI1:4	AK068268	Protein disulfide-isomerase precursor	GAAGCACGCTAGCATCCCTT	GAAGCTGTGCTCTGATCCTTC	She et al (2010)[1]
PDI1:1	AB373950	Protein disulfide-isomerase precursor	AACGATGTGCCAAGCGAGTTCGAT	TTAGAGCTCATCCTTGAGAGGCTC	Kim et al. (2013)[2]
PDI2:3	AK072941	Protein disulfide-isomerase precursor	ATAAGAGGATTTCCAACTATTAAG	TGCTCCTTGATAATCTACTG	Kim et al. (2013)[2]
SMP	AK101770	Seed maturation protein	CTCACCATCCCTGCATTGGT	TTCCATGCTTGTCCACATGC	She et al (2010)[1]
GBSSI	AK070431	Granule-bound starch synthase I	TCCGAGAGGTTCAGGTCATC	ATGAGCTCCTCGGCGTAGTA	She et al (2010)[1]
SSI	AK109458	Starch synthase I	GGCAGTTGATATCAGTGACATATA	AAACTATATGTCACTGATATCAAC	She et al (2010)[1]
SSIIa	AK101978	Starch synthase IIa	GATCGACCAGGATGACGATT	GGGTAAAGCACCTGCAACAT	She et al (2010)[1]
SSIIIa	AK061604	Starch synthase III a	GCCTGCCCTGGACTACATTG	GCAAACATATGTACACGGTTCTGG	She et al (2010)[1]
SSIVa	AK103906	Starch synthase IVa	GGGAGCGGCTCAAACATAAA	CCGTGCACTGACTGCAAAAT	She et al (2010)[1]
Susy3	AK100306	Sucrose synthase 3	CATGTACCCCCTGCTCAACT	GTCAGCTGTAATGCCTGCAA	She et al (2010)[1]
G6PIa	AK068061	Glucose-6-phosphate isomerase, cytosolic B	TCAGCATGCCTACATTCAGC	TGCAACATCCCGAACAAATA	She et al (2010)[1]
AGPL3	AK069296	ADP-glucose pyrophosphorylase large subunit 3	GACCATTTGCGGCAGGAATA	TGGAACAACCAATACCCCAGA	She et al (2010)[1]
AGPS1	AK073146	ADP-glucose pyrophosphorylase small subunit 1	AGAATGCTCGTATTGGAGAAAATG	GGCAGCATGGAATAAACCAC	She et al (2010)[1]
Pull	AB012915	Pullulanase	ACCTTTCTTCCATGCTGG	CAAAGGTCTGAAAGATGGG	She et al (2010)[1]
Amy3B	AK241191	α-amylase 3B	AGCGGTCTCAGAGTTCCTGCA	TCAAATCTTATTCCAGGCACCA	She et al (2010)[1]
Amy3C	AK101358	α-amylase 3C	CTGGCTCCACACAGAACTCA	CGTAGACATCTCCGTCAGCA	She et al (2010)[1]
ISA1	AK060618	Isoamylase	TGCTCAGCTACTCCTCCATCATC	AGGACCGCACAACTTCAACATA	She et al (2010)[1]
SBEI	AK119436	Starch branching enzyme I	GGCATTGCACTCCAAAAGAT	GCTCCAGTTGTTGCCTTCTC	She et al (2010)[1]
SBEIIa	AB023498	Starch branching enzyme IIa	GCCAATGCCAGGAAGATGA	GCGCAACATAGGATGGGTTT	She et al (2010)[1]
Csa	AK110517	Cellulose synthase-like family A	CACGGATCCGATTCCTCCT	TCCAGATCACCCTTTCTTA	She et al (2010)[1]

Supplementary References

1. She, K. C. *et al.* A novel factor *FLOURY ENDOSPERM2* is involved in regulation of rice grain size and starch quality. *Plant Cell* **22**, 3280-3294 (2010).
2. Kim, H. J. *et al.* Effects of Reduced Prolamin on Seed Storage Protein Composition and the Nutritional Quality of Rice. *Int. J. Mol. Sci.* **14**, 17073-17084 (2013).
