# Supplementary material for: Natural variation of OsGluA2 is involved in grain protein content regulation in rice
Source: Nat Commun. 2019 Apr 26;10:1949. doi: 10.1038/s41467-019-09919-y (PMC6486610; doi:10.1038/s41467-019-09919-y)
Supplement: Supplementary file 3 — Description of Additional Supplementary Files [file 41467_2019_9919_MOESM3_ESM.docx]

**Description of Additional Supplementary Files**

File Name: Supplementary Data 1

Description: GPC of 402 rice accessions in two environments and storage protein fraction contents in a subpopulation containing 103 *indica/japonica* cultivars.

File Name: Supplementary Data 2

Description: The geographic information of germplasm used for haplotype analysis.
