# Supplementary material for: Natural variation of OsGluA2 is involved in grain protein content regulation in rice
Source: Nat Commun. 2019 Apr 26;10:1949. doi: 10.1038/s41467-019-09919-y (PMC6486610; doi:10.1038/s41467-019-09919-y)
Supplement: Supplementary file 2 — Peer Review File [file 41467_2019_9919_MOESM2_ESM.pdf]

Reviewers' comments:

Reviewer #1 (Remarks to the Author):

The MS entitled "Natural variation in OsGluA2 plays an important role in
regulating grain protein content in rice" targeted an important issue for rice science what molecular factor and/or gene regulates glutelin contents of rice seed. Although the data in this research covered wide range of experiments needed for isolating the gene corresponding to q-PC1 and for characterizing its expression and interaction with other factors, the construct of the MS is not acceptable as a scientific report. The most defective point is the lack of appealing what are the novel findings. In addition, description in the Discussion is very poor and redundant.
The detailed comments are as follows:

Title: How does the authors define "OsGluA2"? Does OsGluA2 include 5'UTR and 3'UTR?
Line25: The definition of "quality" in this MS is unclear. Here the author touched "nutrition", but in the section Discussion, only quality was written and the authors thought about eating quality as a "quality".

Please clarify the term "quality" by referring the latest review articles by Hori and Umemoto, respectively, published in the book "Rice Genomics, Genetics and Breeding" issued by Springer in 2018. Throughout the MS, the point touched was the molecular-genetic difference of glutelin between indica and japonica.

Line29: Judging from the description in Discussion, q-PC1 is the same one by Peng et al. published in Nature Communications 5:45847 (2014). If this is true, this article should be cited here not as #30.
Line 47: Description of citation is wrong. The authors are Sasaki,T. & Burr,B.

Line 63-65: The reference #8 only described the identification of new glutelin GluD1. A review article describing the grouping glutelins into GluA, GluB, GluC, and GluD should be correctly cited.

Line 89-90: Is the description "We showed that glutelin content is 89 the major contributor to the variation of rice GPC between indica and japonica subspecies" correct? This fact is already written in line 68-74. Please be faithful to write the true fact.

Line 498 (supplementary Table 11, 12 and 13): Please describe how these collections were decided to represent indica/japonica and which institute manages to establish these core collection. In addition, why two sets of collection in Table 11 and 12 were used? How is the geographical distribution of the used rice varieties?

Line 582-584: RFBD is not available from outside China. Readers of this article cannot validate your data. Do the authors cite other public database being installed by the same data in RFBD?

Line 99-133: The results described here have been already known facts, aren't they? Nature Communications needs novel findings. Why all the data here were summarized in Supplemental section? The most key data should be shown in the main text section and additional data is in the supplemental section.

Line 135-145: The data of QTL mapping is the key of this MS. The authors should show the mapping results as a figure, not by a table, with LOD score.

Here, the authors must refer the article described the isolation og q-PC1, even if the previously reported q-PC1 in might not the same as the q^PC1 in this study. Why the authors ignore the previously reported q-PC1?

Line 189: The description "In order to confirm the gene OsGluA2 is the candidate of qPC-10" is incorrect. "In order to confirm the gene OsGluA2 is responsible to qPC-10" is correct.
Line 204: "confirmed that OsGluA2 is the candidate for qPC-10" is also incorrect as described above. The logic " confirm as candidate" is wrong.

Line 222-258: In the report on q-PC1 in Nature Communications published in 2014, the similar pleiotropic effect of responsible gene to q-PC1, OsAAP6 was described. The authors of this MS must know this fact. Considering the situation, the reviewer is very sorry why the authors of this MS did not examine the genetic effect of crossing of NIL-q-PC1 and NIL-q-PC10. Similar description of apparent pleiotropic effects was no more attractive to investigate molecular mechanism of pleiotropic effects.

Line 472-473: Is the description "We previously identified a major QTL qPC-1" correct?. I checked the authors of article on qPC1 and the present MS and revealed no same person and no same affiliation was found. The description "We previously identified a major QTL

qPC-1." must be fake and corrected

Line 476-477: Again. how the the authors define "high-quality rice varirties"?

Reviewer #2 (Remarks to the Author):

In this manuscript, Yang et al. reported that genetic variations in the promoter of OsGluA2 contributed to the grain protein content (GPC) in rice. They identified two environmentally stable QTLs for GPC based on CSSLs derived from indica and japonica cultivars crossing. One stably inherited QTL, qPC-10, was narrowed into 35 kb regions by map-based cloning. Only one gene, which showed expression variation between the two parents and encoded a precursor of Glutelin, was recognized as the candidate gene. The gene function of OsGluA2 was validated by genetic complementary transformation and Cas9-CRISPR. They found that OsGluA2 had pleiotropic effects on rice grain quality and larger protein body II were in GPC enhanced rice. By analyzing OsGluA2 variations in 100 rice accessions, the author concluded that four variants in the promoter were associated with GPC. In addition, the authors tried to investigate the domestication of OsGluA2, and found that no artificial selection was detected around the OsGluA2 locus.
The findings in this paper are of interest and useful to the genetic research and improvement of GPC in rice. Although authors show much data and many analyses have been carried out, I have several concerns that should be considered.

Major concerns:
1. Several haplotypes of OsGluA2 had been identified, but only one haplotype of OsGluA2 from SL431 was transferred into Sasanishiki. It may be also important to transfer the higher expressed haplotypes into other haplotypes. In addition, overexpression of different haplotypes could be tested and added in the result.

2. Natural variation in OsGluA2 had not been fully analyzed in the manuscript. By analyzing the

association between variations in promoter and the GPC, four variations were thought to be related to GPC. However, which one is the crucial variation was still unknown. What's more, many variations were found in the 3005 cultivated rice accessions and 27 wild rice accessions. Were these variations also contributed to the GPC in rice? In fact, in Figure 3b, the types contained the mutation in 3'UTR had more glutelin content than other types. In Figure 3C, type 1 and type 2 were clustered into one group. Why the author concluded that only type 1 was different from other types? It seems that nature variation of OsGluA2 were mainly in the promoter. However, CRISPR was performed in the coding region. Therefore, CRISPR or other experiments on the promoter could be added in the result to identify the causative variants.

3. The samples used in the research and the division of rice germplasm were confused throughout the manuscript. In the glutelin content detection, the 404 rice cultivars were divided into indica and japonica. In the four components of storage protein detection, 60 indica and 44 japonica cultivars were used. In the variation detection of OsGluA2, 100 accessions were clustered into temperate japonica, tropical japonica, aus, indica and aromatic. In the evolution analysis, 3005 cultivated rice accessions were divided into seven groups. The author should use all the 404 rice cultivars in both the sequence variation analysis and four components protein detection. In addition, the division of all samples should be unified.

4. The author tried to demonstrate that the variation of OsGluA2 between indica and japonica was the main reason for the GPC variation. However, the data they used in the related analyses are defective. The authors analyzed the GPC in 404 rice accessions, including 204 indica and 200 japonica cultivars. However, I did not find any explanation that how these accessions were divided into indica and japonica. For example, one famous variety belonged to aromatic (basmati lines) was divided into indica in the manuscript (it actually belong to japonica subgroup). Genome sequence, phenotypic and physiologic data should be given to support the division of the accessions. In Fig. 3b, proportion of type 1 (OsGluA2LET) in Tej, Trj, Aus, Ind, and Aro was 74%, 16%, 0%, 9% and 100%, respectively. The proportion of type 1 in tropical japonica seems to be not in line with the conclusion.

5. There were many mistakes in the figures. In Fig. 1a, the marker names were not in direct line and the width of dotted lines were different. In Fig. 1b, the protein content of the fifth line was missing and the mean value of the sixth line was not indicated. In Figure 2a, the tissues were not displayed, but the description was in the figure legend. The physical locations of the variations and small lines indicating ATG and TAA in Figure 3a were not in the right positions. Moreover, usually CDS do not include intron regions. However, CDS in Figure 3a contained intron regions. The explanation of 'a' and 'b' in Figure 3d was not found in figure legend. The description of '*' in Figure 4d was missing. Figure legend 4 mentioned that "Black spots indicated unidentified haplotypes". However, the bar in Figure 4f indicated that black spots were wild accessions or OsGluA2HET haplotypes, which may lead to confusions.

6. Geographic origin of the rice accessions had not been provided in supplementary table 11 and table 12.

7. In line 152-153, the author mentioned 'SDS-PAGE 152 analysis showed that SL431 has higher accumulation of 57-KD proglutelins, acidic 153 and basic subunits and prolamins than Sasanishiki'. However, no significantly different protein belt in 57-KD was found in Supplementary Figure 2c.

8. Figure 2a showed OsGluA2 in NIL-qPC-10H and NIL-qPC-10S had different expressions in 25 days after flowering. However, GUS only showed the results of 15 days and 20 days after flowering in Figure 2b and 2c. It is better to add the GUS result of 25 days after flowering.

9. In total, 3005 cultivated rice accessions from all around the world were used in the domestication analysis, but no wild rice accessions were used. However, for detecting domestication signals, the O. rufipogon samples are necessary. Hence, this result did not provide any solid information.

10. In line 251, page 12, the author mentioned that 16 key genes were selected and the transcription were examined. Why the author selected these genes? Why there were 32 genes showed in Figure 2d and some of them were starch synthetic genes but not protein synthetic genes.

11. Map-based cloning should be descripted in the method section.

Minor concerns:
1. Usually, 'And' did not use in the beginning of sentences. 'And' in line 28 and line 105 should be changed.

2. In line 72, the reference 71 appeared before reference 12.

3. 'decades' in line 75 should be decade.

4. 'the' in line 86 should be removed.

5. What does 'mini-core germplasms' in ine 101 mean? In general, mini-core collections were used by the scientists that focus on germplasm resources. It is referred to a small quantity of germplasms that selected from the core collections and could represent the genetic diversity of most samples, including both landraces and varieties. Is these 'mini-core germplasm' from the USDA or IRRI? If not, 'mini-core' should not be used here.

6. Many blank spaces that before and after the operational characters and units were missing in the manuscript. For example, '8~11%' in line 104, '11.17% ± 1.15%' in line 111, 'p=5.7×10-12' in line 127, '~80kb' in line 592, '2000bp' in line 593. Please carefully check.

7. The 'P' in line 184 and line 156 were in capital but in line 236 and line 237 was in lower case.

8. There were extra spaces in 'NIL- qPC-10s' in line 255 and 'NIL- qPC-10H' line 265. In addition, the 'S' in 'NIL- qPC-10s' in line 255 was in lower case. These should be corrected.

9. It is 'kb' in line 192, but 'Kb' in line 147, line173 and line 274.

10. One space before UTR should be added in '3'UTR' in line 275.

11. There were extra spaces in '（Glb-1）' in line 457.

12. I suppose that 'Haina' in Supplementary table 3 should be 'Hainan'.

13. Detail of the near-infrared spectroscopy method should be descripted in the method section. For example, how many samples had been used in the model construction? Which kind of samples was used, milled rice, polished rice or unpolished rice?

14. Why the haplotypes with less than six rice accessions were remove from Figure 4f? It should be explanted.

15. It is hard to understand the sentence in line 420-422. It needs to be revised.

16. In line 478, LGC-1 mutant and W3660 were mentioned. However, the glutelin contents of these cultivars were not provided.

17. Some of the journal names in the reference were full name, but the others were abbreviated name.

18. ‘FLOURY ENDOSPERM2’ in reference 19 (line 656) should be italic.

19. Page number of reference 12 (line 641-642) was not provided.

20. The comma was missing in ‘16 000rpm’ in ine 572. In addition, one space was also missing.

Reviewer #3 (Remarks to the Author):

This paper reports the identification of a locus, Gpc-10, that influences grain protein content. The authors show that the locus encodes a glutelin, OsGlu-A2 that is generally expressed more highly in the Indica subspecies than Japonica. CRISPR knockouts and over-expression are used to show that the expression of the gene does influence grain protein content. The authors provide comprehensive data.

I feel that the authors have uncovered the hints of an intriguing story. Based on their results and without preconceptions of what the OsGlu-A2 gene does, it would seem that this is like a transcription factor/RNA regulator that up-regulates the transcription of a number of storage protein genes. The authors point out (lines 228-231), the increased protein content is not just due to increased accumulation of OsGlu-A2 itself but generalized increase of protein accumulation. So somehow this increased expression of OsGlu-A2 is triggering wide-spread change in protein expression. Some of the changes in the transcription is far greater than shown by OsGlu-A2 itself. For example the transcription of Os-GluA1 according to Fig 2d is increased about three fold.

I would suggest the authors consider the following to make the story clearer.

1. Is it possible that some small RNAs are being transcribed from this same region that is influencing transcription of a large number of genes? In that case the OsGlu-A2 gene is almost a by-stander. Is it possible to do a sequence comparison of the small RNAs in the paired knockout and over-expressing lines?

2. The paper does not directly show how much greater OsGlu-A2 protein accumulation is. There is the data on the expression at the RNA level but no qualitative or quantitative assay of OsGlu-A2 protein. Is it in fact accumulating more? Is it not possible to use antibodies/Western blotting to show directly that the increased transcription of the RNA is leading to more of the OsGlu-A2 protein accumulation? The results from this may indicate mechanisms by which the general increase is taking place.

Minor points

1. In general the paper reads well although some editing is required of the English. For example it should be ‘chose’ not ‘choose’ in line 198 and some other sentence constructions (starting with And etc) left me a little uncomfortable.

2. In Fig 2c the colouring is wrong and reverse of what it should be.

3. It would be good to have a Table with list of the names of the genes being assayed in Fig2d. This could go in the supplementary data. For example I cannot immediately find out what SMP is.

Reviewers' comments:

Reviewer #1

The MS entitled "Natural variation in OsGluA2 plays an important role in regulating grain protein content in rice" targeted an important issue for rice science what molecular factor and/or gene regulates glutelin contents of rice seed. Although the data in this research covered wide range of experiments needed for isolating the gene corresponding to q-PC1 (actually *qPC-10*) and for characterizing its expression and interaction with other factors, the construct of the MS is not acceptable as a scientific report. The most defective point is the lack of appealing what are the novel findings. In addition, description in the Discussion is very poor and redundant.

Response: Thank you for your comments. Following your suggestions, we have restructured the MS and modified the description of discussion after a deep reconsideration, please check it. To our knowledge, grain protein content (GPC) in rice endosperm plays an important role in determining rice grain quality, associated with both nutritive value and palatability. As for novel findings, we considered that the highlight of present study includes:

1) We firstly revealed that glutelin content is a key factor contributing to GPC variation in rice germplasm and acts as a main reason causing the GPC variation between the *indica* and *japonica* subspecies.
2) GPC belongs to a typical quantitative trait with complex genetic structure and sensibility to environmental factors, especially to the amount of nitrogen fertilizer application at late growing stages. Therefore, the QTLs for rice GPC detected using different genetic populations are frequently inconsistent, and few of them can be detected repeatedly. To date, only one QTL has successfully been cloned. In our work, a major QTL, *qPC-10* (*OsGluA2*) controlling GPC, was successfully cloned and functionally characterized, which provides gene resource for genetic improvement in rice GPC.
3) We revealed that the differentiation on *OsGluA2* is associated with the *indica/japonica* differentiation. Two functional types were identified as $OsGluA2^{LET}$ and $OsGluA2^{HET}$, and the former is originated from wild species.
4) The cloned *OsGluA2* could be used as the target for rice quality improvement through molecular marker-aided selection or gene editing strategy.

Taken together, our findings bring new insights into our understanding of GPC variation and may benefit rice breeding.

The detailed comments are as follows:

Title: How does the authors define "OsGluA2"? Does OsGluA2 include 5'UTR and 3'UTR?

Response: Sorry for this confusion. The *OsGluA2* (GeneBank ID: Y00687) includes 5' UTR and 3' UTR in MS.

Line25: The definition of "quality" in this MS is unclear. Here the author touched "nutrition", but in the section Discussion, only quality was written and the authors thought about eating quality as a "quality". Please clarify the term "quality" by referring the latest review articles

by Hori and Umemoto, respectively, published in the book “Rice Genomics, Genetics and Breeding” issued by Springer in 2018. Throughout the MS, the point touched was the molecular-genetic difference of glutelin between indica and japonica.

Response: Thank you for underlining this deficiency and your introduction of the latest reviews in the book “Rice Genomics, Genetics and Breeding”. GPC in rice endosperm plays an important role in determining rice grain quality, especially the nutrition quality and eating and cooking quality. Glutelin accounts for as much as 60 – 80 % of the total GPC. So, any change of glutelin content will certainly affect the grain quality. In our work, a major QTL, *qPC-10,* encoding a glutelin type-A2 precursor OsGluA2, which functions as a positive regulator of GPC in rice, was successfully cloned and functionally characterized. Following your suggestions, we have redefined the “quality” by referring the latest review articles by Hori and Umemoto and further described relationship among glutelin content, GPC and grain quality in the revised MS, please check it.

Line29: Judging from the description in Discussion, q-PC1 is the same one by Peng et al. published in Nature Communications 5:45847 (2014). If this is true, this article should be cited here not as #30.

Response: Actually, the *qPC-1* in Line 29 is not the same as *qPC1* published in Nature Communication 5:45847 (2014). We previously identified the major QTL *qPC-1* which was delimited to a 41-Kb DNA region between the marker W3 and W5 on Chromosome 1 in 2015, which is the same locus in the present study. However, *qPC1* has been cloned (Peng et al. 2014), encoding a putative amino acid permease, *OsAAP6*, and was found not to be in the *qPC-1*-anchored region. Hence, we are sure that *qPC-1* identified in the present study is nonallelic to *qPC1* identified in the cross of Zhenshan 97/Nanyangzhan (Peng et al. 2014), and *qPC-1* may regulate the GPC in a different way. please check the ref 28 and ref 35.

Line 47: Description of citation is wrong. The authors are Sasaki,T. & Burr,B.

Response: We are very sorry for our negligence. The mistake has been corrected, please check Line 587.

Line 63-65: The reference #8 only described the identification of new glutelin GluD1. A review article describing the grouping glutelins into GluA, GluB, GluC, and GluD should be correctly cited.

Response: Thank you for pointing this out. Following your suggestion, we have cited review article to replace the previous one, please check ref 13.

Line 89-90: Is the description “We showed that glutelin content is 89 the major contributor to the variation of rice GPC between indica and japonica subspecies” correct? This fact is already written in line 68-74. Please be faithful to write the true fact.

Response: Thank you for your comment. Previous researches in Line 68-74 just revealed that the rice GPC differences between two subspecies. But they did not explain why the GPC in *indica* subspecies is generally higher than that in *japonica*. The truth is that GPC consists of functional protein (~ 10%) and storage proteins which account for ~ 90% of

GPC. The storage proteins have four categories, including albumins, globulins, prolamins and glutelins. Among them, glutelin is the most abundant and comprises about 60 - 80% of the total storage proteins. In our work, we further assayed the contents of four components of storage protein in 43 representative *indica* and 60 *japonica* cultivars and firstly revealed that the variation of rice glutelin content is the key factor in determining the GPC variation, accounting for more than 70% (R^2 = 0.739) of total GPC variation (**Fig. 1c** in the revised manuscript). Therefore, we think that the description in Line 89-90 is not a contradiction for the representation in Line 68-74.

Line 498 (supplementary Table 11, 12 and 13): Please describe how these collections were decided to represent indica/japonica and which institute manages to establish these core collection. In addition, why two sets of collection in Table 11 and 12 were used? How is the geographical distribution of the used rice varieties?

Response: Thank you for pointing these out. We have rechecked the division of these collections (Zhang et al. 2011, Wang et al. 2018) and reanalyzed the data in the revised MS. Actually, these rice accessions used in our work were selected from a large collection of rice cultivars from China National Rice Research Institute and consisted of landrace varieties (LV, pureline varieties developed by farmers without artificial intercrossing) and modern pureline varieties (MPV) in China. According to the records of the Chinese Crop Germplasm Information Network (http://icgr.caas.net.cn/pztest.html), all accessions could be allocated to two subspecies (*indica* and *japonica*) (Zhang et al. 2011). At the same time, most of the these rice accessions have already been sequenced and population structure analyzed (Wang et al. 2018). In our work, the set of collection in Supplementary Table 12 is actually the subpopulation of rice accessions in Supplementary Table 11. As we know, GPC is a typical quantitative trait, many factors have been shown to affect GPC, such as plant height and heading date. In order to minimize the interference of the above factors, we chose 103 rice cultivars with similar plant height and heading date to develop the subpopulation. Therefore, to resolve the confusion, we have combined these two tables and added the geographical distribution of them (**Supplementary Table 9** in the revised manuscript).

It should be noted that, in the original MS, we described that 410 accessions in total were used to survey the GPC, but we found some of them are incorrectly classified and repetitive, so in the revised MS, we moved them out, and the data were reanalyzed.

Zhang, H. *et al*. A core collection and mini core collection of Oryza sativa L. in China. *Theor. Appl. Genet*. **122**,49–61 (2011).

Wang, W. Sh. *et al*. Genomic variation in 3,010 diverse accessions of Asian cultivated rice. *Nature* **557**, 43-49 (2018).

Line 582-584: RFBD is not available from outside China. Readers of this article cannot validate your data. Do the authors cite other public database being installed by the same data in RFBD?

Response: Many thanks for your comments. According to the annotation of the database,

we have change the abbreviation of Rice Functional Genomics and Breeding Database to RFGB. In this database, the genomic information of about 3,000 rice accessions (3K rice genome) can be obtained. We further noticed that some browsers can not open this website through the link http://www.rmbreeding.cn/. In the revised manuscript, the link have been changed to http://www.rmbreeding.cn/Snp3k. In addition, more information of 3K rice genomes can also be found through the link http://www.rmbreeding.cn/Index/. We have tested the website in Canada and USA, and found that the last two links can be opened directly.

Line 99-133: The results described here have been already known facts, aren't they? Nature Communications needs novel findings. Why all the data here were summarized in Supplemental section? The most key data should be shown in the main text section and additional data is in the supplemental section.

Response: Thank you for underlining this deficiency. We totally agree with your comments and have restructured the MS after deep reconsideration, please check it. In the revised MS, we added a new figure, as **Figure 1**, in the revised MS for describing the result.

Line 135-145: The data of QTL mapping is the key of this MS. The authors should show the mapping results as a figure, not by a table, with LOD score. Here, the authors must refer the article described the isolation og q-PC1, even if the previously reported q-PC1 in might not the same as the q^PC1 in this study. Why the authors ignore the previously reported q-PC1?

Response: Thank you for your comments. Following your suggestions, we have showed the mapping results as a figure with LOD score, please check the **Fig. 2a** in revised MS. As the *qPC-1* here is not the same as *qPC1* published in Nature Communication 5:45847 (2014), we think that there is no need to refer it.

Line 189: The description "In order to confirm the gene OsGluA2 is the candidate of qPC-10" is incorrect. "In order to confirm the gene OsGluA2 is responsible to qPC-10" is correct.

Response: Thank you for pointing this out. According to your suggestion, the sentence "In order to confirm the gene *OsGluA2* is the candidate of *qPC-10*" was revised to "In order to confirm the gene *OsGluA2* is responsible to *qPC-10*" in the revised manuscript in Line 185.

Line 204: "confirmed that OsGluA2 is the candidate for qPC-10" is also incorrect as described above. The logic " confirm as candidate" is wrong.

Response: Thank you for pointing this logical error out. According to your suggestion, the sentence "confirmed that *OsGluA2* is the candidate for qPC-10" was revised to "confirmed that *OsGluA2* is responsible to *qPC-10*" in the revised manuscript in Line 199-200.

Line 222-258: In the report on q-PC1 in Nature Communications published in 2014, the similar pleiotropic effect of responsible gene to q-PC1, OsAAP6 was described. The authors of this MS must know this fact. Considering the situation, the reviewer is very sorry why the authors of this MS did not examine the genetic effect of crossing of NIL-q-PC1 and

NIL-q-PC10. Similar description of apparent pleiotropic effects was no more attractive to investigate molecular mechanism of pleiotropic effects.

Response: Thank you for your constructive suggestion. We guess the reviewer's suggestion is to examine the genetic effect of the crossing of NIL-qPC1(*OsAAP6*) and NIL-qPC-10. In fact, *qPC1* (*OsAAP6*) was cloned by Peng et al. (2014), not by our team. In their work, the NIL was generated with *indica* parent as genetic background. In contrast, Sasanshiki, a *japonica* parent was selected as genetic background for developing NIL for *OsGluA2*. So, if cross the two set of NILs, the progeny will dramatically segregate in many aspects, which will hind us to evaluate the genetic effect of *qPC1* (*OsAAP6*) and *qPC-10* (*OsGluA2*). Thus, continue backcrossing are needed, it is a time-consuming work. On the other hand, it was reported that the expression level of *OsAAP6* is found to be associated with GPC variation mainly in *indica* cultivars, whereas there is no significant difference in GPC in *japonica* backgrounds, that is, even though the *OsAAP6* was pyramided with *OsGluA2* in the *japonica* background, it will not yield a meaningful result.

However, if reviewer's suggestion is to examine the genetic effect of crossing of NIL-*qPC-1* (identified in the present study) and NIL-*qPC-10*, we fully agree with reviewer's idea and think it was worth to do that. However, it will need time for developing the NILs and the phenotype analysis, at least 1-2 years will be needed in rice. Considering the timeliness of the research article, we will seriously take your advice and show the evidences in the future studies on the genetic effect of crossing of NIL-*qPC-1* and NIL-*qPC-10*.

Line 472-473: Is the description "We previously identified a major QTL qPC-1" correct? I checked the authors of article on qPC1 and the present MS and revealed no same person and no same affiliation was found. The description "We previously identified a major QTL qPC-1." must be fake and corrected

Response: I am sorry for making it unclear in MS. The *qPC-1* and *qPC1* are different locus. The *qPC-1* was identified by Yang et al. in 2015 (published in *Molecular Breeding*), whereas, *qPC1* was cloned by Peng et al. in 2014 (published in *Nature Communications*). Please check the ref 28 and ref 35.

In fact, before we prepared the paper about the fine-mapping of *qPC-1*, Peng *et al*. has published their work. But we also identified a QTL on chromosome 1, we named it as "*qPC-1"*, inserting a dash between the character and number in order to distinguish "*qPC1*" identified by Peng *et al*. We are very sorry for making the reviewer confused.

Line 476-477: Again. how the authors define "high-quality rice varieties"?

Response: To be honest, it is difficulty for researchers to define "high quality rice varieties", because "quality" is a comprehensive character which consists four major components, concerning grain appearance, milling qualities, nutrition, and cooking and eating quality. As to GPC of rice, it is well known that higher GPC means higher nutritional quality. However, high GPC may lead to densely structured rice grains, which will result in hard and loose cooked rice and, thus, poor palatability. Therefore, to address this concern, we have reworded the sentence to our statement (Line 347-351).

Reviewer #2 (Remarks to the Author):

In this manuscript, Yang et al. reported that genetic variations in the promoter of OsGluA2 contributed to the grain protein content (GPC) in rice. They identified two environmentally stable QTLs for GPC based on CSSLs derived from indica and japonica cultivars crossing. One stably inherited QTL, qPC-10, was narrowed into 35 kb regions by map-based cloning. Only one gene, which showed expression variation between the two parents and encoded a precursor of Glutelin, was recognized as the candidate gene. The gene function of OsGluA2 was validated by genetic complementary transformation and Cas9-CRISPR. They found that OsGluA2 had pleiotropic effects on rice grain quality and larger protein body II were in GPC enhanced rice. By analyzing OsGluA2 variations in 100 rice accessions, the author concluded that four variants in the promoter were associated with GPC. In addition, the authors tried to investigate the domestication of OsGluA2, and found that no artificial selection was detected around the OsGluA2 locus.
The findings in this paper are of interest and useful to the genetic research and improvement of GPC in rice. Although authors show much data and many analyses have been carried out, I have several concerns that should be considered.
Response: Thank you for the positive comments.

Major concerns:
1. Several haplotypes of OsGluA2 had been identified, but only one haplotype of OsGluA2 from SL431 was transferred into Sasanishiki. It may be also important to transfer the higher expressed haplotypes into other haplotypes. In addition, overexpression of different haplotypes could be tested and added in the result.
Response: Thank you for your kind suggestions. In our work, based on the transcript abundance and the glutelin content of *OsGluA2* of five haplotypes, we divided these five haplotypes into two types, low expression type ($OsGluA2^{LET}$) which includes Haplotype 1 and higher expression type ($OsGluA2^{HET}$) which contains Hap 2-5. The cultivars possessing haplotype1 tended to show significant lower expression levels in endosperms and a lower glutelin content (**Fig.4d** and **4e** in the revised manuscript), while the other four haplotypes show similar expression levels and glutelin content with no statistically significant difference. At the same time, we have transferred the haplotype 4 (SL431) into the haplotype1 (Sasanishiki) and transgene-positive plants showed a significant increase in the GPC. Therefore, we have reasons to believe that it will not make any new findings when transfer hap2-hap5 into hap1 or be transferred by each other.

Many researches have shown that the promoters of *SSPs* gene are highly and specifically expressed active in the endosperm tissue and are used for production of recombinant proteins in rice seeds (Qu et al, 2004; Yang et al, 2006). Their drive capacity is strong, which is equivalent to 35S promoter to some extent. Therefore, we have reason to believe that the results of complementation test are enough.

Qu, L.Q. & Takaiwa, F. Evaluation of tissue specificity and expression strength of rice seed component gene promoters in transgenic rice. *Plant Biotechnol. J.* **2**, 113–125 (2004).

Yang, L. J. *et al.* A transgenic rice seed accumulating an anti-hypertensive peptide reduces the blood pressure of spontaneously hypertensive rats. *FEBS Letters* **580,** 3315-3320 (2006)

2. Natural variation in OsGluA2 had not been fully analyzed in the manuscript. By analyzing the association between variations in promoter and the GPC, four variations were thought to be related to GPC. However, which one is the crucial variation was still unknown. What's more, many variations were found in the 3005 cultivated rice accessions and 27 wild rice accessions. Were these variations also contributed to the GPC in rice? In fact, in Figure 3b, the types contained the mutation in 3'UTR had more glutelin content than other types. In Figure 3C, type 1 and type 2 were clustered into one group. Why the author concluded that only type 1 was different from other types? It seems that nature variation of OsGluA2 were mainly in the promoter. However, CRISPR was performed in the coding region. Therefore, CRISPR or other experiments on the promoter could be added in the result to identify the causative variants.

Response: Thank you very much for your kind suggestions. We totally agree with you that it is important to find out which SNPs in the promotor are crucial to the variation of GPC. Following your suggestion, we conducted transient assays of the site-directed mutated promoter fragments of *OsGluA2* in rice protoplasts to test the effects of the four SNPs in the promoter region. Compared with the activity of the Sasanishiki promoter fragment, the relative activities of the promoter fragments with one (M3, -1100 position) mutation was greatly increased ($P \leq 0.05$) (**Fig. 4f** in the revised manuscript). These results indicated that the SNP - 1100 might be functional SNP (FNP) accounting for the expression level differences between the *OsGluA2* alleles. We added the sentences in revised MS, please check Line 285-296.

Thank you for your feedback. Based on the difference of genomic sequence of these five haplotypes, type1 and type2 were clustered into one group in figure 4c. However, in terms of transcript abundance of *OsGluA2* of these five types, the cultivars possessing type 1 showed significant lower expression levels in endosperms at 15 DAF and a lower glutelin content than other types. So, we concluded that only type 1 was different from other types. In order to resolve your confusion, we added the sentences in revised MS, please check Line 278.

3. The samples used in the research and the division of rice germplasm were confused throughout the manuscript. In the glutelin content detection, the 404 rice cultivars were divided into indica and japonica. In the four components of storage protein detection, 60 indica and 44 japonica cultivars were used. In the variation detection of OsGluA2, 100 accessions were clustered into temperate japonica, tropical japonica, aus, indica and aromatic. In the evolution analysis, 3005 cultivated rice accessions were divided into seven groups. The author should use all the 404 rice cultivars in both the sequence variation analysis and four components protein detection. In addition, the division of all samples should be unified.

Response: Thank you for pointing out the deficiency. We did not used the same sample in our work, mainly due to the different purposes. In our work, for GPC survey in rice

germplasm, a big population, in total of 402 *indica/japonica* accessions (listed in Supplementary Table 11) were used. For SSP fraction analysis, a relatively small population consisting of 103 accession was used (Supplementary Table 12), which is selected from the core collection of 410 rice accessions listed in Supplementary Table 11. Because GPC is a typical quantitative trait, many factors have been shown to affect GPC, such as plant height and heading date. In order to minimize the interference of the above factors, we chose 103 rice cultivars from core collection with similar plant height and heading date to generate the subpopulation. Therefore, to resolve the confusion, we have merged these two tables, added the geographical distribution of them in **Supplementary Table 9** in the revised manuscript, and Supplementary Table 12 was moved out.

In addition, as we know, Asian cultivated rice contains two main subspecies, *japonica* and *indcia*. However, in recent years, based on the genome sequence information, Asian cultivated rice could be divided into at least five ecotype, e.g. *indica*, *Aus*, Aromatic, tropical *japonica* and temperate *japonica* (Zhao et al. Nature Communications, 2011). Among them, ecotype *indica*, *Aus* and *Aromatic* belong to *indica* subspecies, and ecotype temperate and tropical *japonica* belong to *japonica* subspecies. In the present study, for the purpose of investigation of GPC in rice germplasm, 402 accessions were used, most of them are typical *indica* and *japonica* cultivars from China, mainly consisting of two ecotypes, *indica* and *temperate japonica*. We think this collection is not qualified for studying the gene variation due to its small genetic information, so, a subpopulation from a diversity panel consisting of 413 accessions from 82 countries (Zhao et al., 2011) was employed to study *OsGluA2* natural variation. Similarly, a big population containing ~3000 accessions (Wang et al. 2018) was used to trace the evolution of *OsGluA2*. Both of these two population consisting of at least five ecotypes. Thus, the division of samples used in natural variation analysis and evolution of *OsGluA2* is unified.

Zhao, K. et al. Genome-wide association mapping reveals a rich genetic architecture of complex traits in *Oryza sativa. Nature Communications* 2:467 (2011)

Wang, W. Sh. *et al*. Genomic variation in 3,010 diverse accessions of Asian cultivated rice. *Nature* **557**, 43-49 (2018).

4. The author tried to demonstrate that the variation of OsGluA2 between indica and japonica was the main reason for the GPC variation. However, the data they used in the related analyses are defective. The authors analyzed the GPC in 404 rice accessions, including 204 indica and 200 japonica cultivars. However, I did not find any explanation that how these accessions were divided into indica and japonica. For example, one famous variety belonged to aromatic (basmati lines) was divided into indica in the manuscript (it actually belong to japonica subgroup). Genome sequence, phenotypic and physiologic data should be given to support the division of the accessions. In Fig. 3b, proportion of type 1 (OsGluA2LET) in Tej, Trj, Aus, Ind, and Aro was 74%, 16%, 0%, 9% and 100%, respectively. The proportion of type 1 in tropical japonica seems to be not in line with the conclusion.

Response: Thank you for pointing out the deficiency. We have rechecked the division of

these collections (Zhang et al. 2011, Wang et al. 2018) and reanalyzed the data in the revised MS. As for Basmati (Aromatic ecotype), they generally were bred and cultivated in India, Thailand and Pakistan, where are tropical area fitting for cultivating *indica* accessions. In fact, most of rice scientists think Basmati rice belong to *indica* subspecies. For example, Wang et al. used 25 basmati lines to understand the variations on *SPL16* (*GW8*), they divided them into *indica* group (detailed see Supplementary Table 5 in Nature Genetics 3522, 2015).

As for the distribution of $OsGluA2^{LET}$ in population, we also noticed that the proportion of type1 ($OsGluA2^{LET}$) is relatively low (16%). Coincidently, similar result was also observed in the population with 3,005 accessions, the proportion of type 1 ($OsGluA2^{LET}$) is ~36% (141/387) (**Supplementary Table 8** in the revised manuscript) in tropical *japonica* accessions. In the revised MS, we just concluded that $OsGluA2^{LET}$ mainly existed in temperate japonica.

Zhang, H. *et al*. A core collection and mini core collection of Oryza sativa L. in China. *Theor. Appl. Genet*. **122**,49–61 (2011).
Wang, W. Sh. *et al*. Genomic variation in 3,010 diverse accessions of Asian cultivated rice. *Nature* **557**, 43-49 (2018).
Wang S.H. *et al.* The OsSPL16-GW7 regulatory module determines grain shape and simultaneously improves rice yield and grain quality. *Nature Genet.* **47**, 949-954 (2015)

5. There were many mistakes in the figures. In Fig. 1a, the marker names were not in direct line and the width of dotted lines were different. In Fig. 1b, the protein content of the fifth line was missing and the mean value of the sixth line was not indicated. In Figure 2a, the tissues were not displayed, but the description was in the figure legend. The physical locations of the variations and small lines indicating ATG and TAA in Figure 3a were not in the right positions. Moreover, usually CDS do not include intron regions. However, CDS in Figure 3a contained intron regions. The explanation of ‘a’ and ‘b’ in Figure 3d was not found in figure legend. The description of ‘*’ in Figure 4d was missing. Figure legend 4 mentioned that “Black spots indicated unidentified haplotypes”. However, the bar in Figure 4f indicated that black spots were wild accessions or OsGluA2HET haplotypes, which may lead to confusions.

Response: We very much appreciate your careful reading of our manuscript. Following your suggestions, all the mistakes in the figures have been corrected, please check it.

6. Geographic origin of the rice accessions had not been provided in supplementary table 11 and table 12.

Response: In order to resolve the confusion, we have combined these two tables together and added the geographical distribution of them (**Supplementary Table 9 and 10** in the revised manuscript).

7. In line 152-153, the author mentioned ‘SDS-PAGE 152 analysis showed that SL431 has higher accumulation of 57-KD proglutelins, acidic 153 and basic subunits and prolamins

than Sasanishiki'. However, no significantly different protein belt in 57-KD was found in Supplementary Figure 2c.

Response: Thank you very much for comments. There does not seem to make much difference in 57 KD at first glance. However, 57 KD precursor can cleave into a 37-39 KD acidic subunit and a 22-23 KD basic subunit in cytoplasm. Put them all together, SDS-PAGE analysis indeed showed that SL431 has higher accumulation of glutelin content than Sasanishiki. To resolve this confusion, the densitometry of the gels was measured by using the Image J software. The result showed that SL431 has significant higher accumulation of 57-KD proglutelins, acidic and basic subunits and prolamins than Sasanishiki (**Supplementary Fig 1c** in the revised manuscript).

8. Figure 2a showed OsGluA2 in NIL-qPC-10H and NIL-qPC-10S had different expressions in 25 days after flowering. However, GUS only showed the results of 15 days and 20 days after flowering in Figure 2b and 2c. It is better to add the GUS result of 25 days after flowering.

Response: Thank you for pointing it out. Following your suggestion, we added the GUS result of 25 days after flowering in revised MS (**Fig. 3b and 3c** in the revised manuscript).

9. In total, 3005 cultivated rice accessions from all around the world were used in the domestication analysis, but no wild rice accessions were used. However, for detecting domestication signals, the *O. rufipogon* samples are necessary. Hence, this result did not provide any solid information.

Response: Many thanks for your suggestion. To determine the putative domestication signals in *OsGluA2*, we used the genomic information of 3,005 cultivated rice and 27 *O. rufipogon* accessions to analyze the genetic diversity of this gene and its flanking regions. The results can be found in **Fig. 5c-f** in the revised manuscript.

According to your suggestion, we have changed the sentence to "In order to reveal whether artificial selection have been contributed to the domestication of *OsGluA2*, the genomic information of 3005 cultivated rice and 27 *O. rufipogon* accessions were used to analyze the genetic diversity of this gene and its flanking regions."

10. In line 251, page 12, the author mentioned that 16 key genes were selected and the transcription were examined. Why the author selected these genes? Why there were 32 genes showed in Figure 2d and some of them were starch synthetic genes but not protein synthetic genes.

Response: Because we observed that prolamin and glutelin contents altered in the NIL and transgenic lines, we examined the genes transcription levels, these 16 genes have been confirmed to be involved in protein synthesis. Similarly, in order to understand why the starch composition and physic-chemical properties changes, 16 starch synthesis related genes also were selected subject to measurement of their transcription statues. These genes information is listed in the **Supplementary Table 12** in the revised manuscript.

11. Map-based cloning should be descripted in the method section.

Response: Thank you for your suggestion, we have descripted Map-based cloning in the

method section, please check the Line 451-457.

Minor concerns:

1. Usually, ‘And’ did not use in the beginning of sentences. ‘And’ in line 28 and line 105 should be changed.

Response: Sorry for the mistake, we have corrected these mistakes in the revised MS.

2. In line 72, the reference 71 appeared before reference 12.

Response: We very much appreciate your careful reading of our manuscript, and it has been corrected in the revised manuscript.

3. ‘decades’ in line 75 should be decade.

Response: We very much appreciate your careful reading of our manuscript, and it has been corrected in the revised manuscript.

4. ‘the’ in line 86 should be removed.

Response: We very much appreciate your careful reading of our manuscript, and it has been corrected in the revised manuscript.

5. What does ‘mini-core germplasms’ in line 101 mean? In general, mini-core collections were used by the scientists that focus on germplasm resources. It is referred to a small quantity of germplasms that selected from the core collections and could represent the genetic diversity of most samples, including both landraces and varieties. Is these ‘mini-core germplasm’ from the USDA or IRRI? If not, ‘mini-core’ should not be used here.

Response: Thank you for pointing these out. The 402 rice accessions used in our work were selected from a large collection of rice cultivars from China National Rice Research Institute and consisted of landrace varieties (LV, pureline varieties developed by farmers without artificial intercrossing) and modern pureline varieties (MPV) in China. In order to resolve the confusion, we have removed the “mini-core” and reworded the sentence in line 101.

6. Many blank spaces that before and after the operational characters and units were missing in the manuscript. For example, ‘8~11%’ in line 104, ‘11.17% ± 1.15%’ in line 111, ‘p=5.7×10-12’ in line 127, ‘~80kb’ in line 592, ‘2000bp’ in line 593. Please carefully check.

Response: We very much appreciate your careful reading of our manuscript, and it has been corrected in the revised manuscript.

7. The ‘P’ in line 184 and line 156 were in capital but in line 236 and line 237 was in lower case.

Response: We very much appreciate your careful reading of our manuscript, and it has been corrected in the revised manuscript.

8. There were extra spaces in ‘NIL- qPC-10s’ in line 255 and ‘NIL- qPC-10H‘ line 265. In addition, the ‘S’ in ‘NIL- qPC-10s’ in line 255 was in lower case. These should be corrected.

Response: We very much appreciate your careful reading of our manuscript, and it has been corrected in the revised manuscript.

9. It is ‘kb’ in line 192, but ‘Kb’ in line 147, line173 and line 274.

Response: We very much appreciate your careful reading of our manuscript, and it has been corrected in the revised manuscript.

10. One space before UTR should be added in ‘3’UTR’ in line 275.

Response: We very much appreciate your careful reading of our manuscript, and it has been corrected in the revised manuscript.

11. There were extra spaces in ‘（Glb-1）’ in line 457.

Response: We very much appreciate your careful reading of our manuscript, and it has been corrected in the revised manuscript.

12. I suppose that ‘Haina’ in Supplementary table 3 should be ‘Hainan’.

Response: We very much appreciate your careful reading of our manuscript, and it has been corrected in the revised manuscript.

13. Detail of the near-infrared spectroscopy method should be descripted in the method section. For example, how many samples had been used in the model construction? Which kind of samples was used, milled rice, polished rice or unpolished rice?

Response: Thank you for your suggestion, we have descripted detail of the near-infrared spectroscopy method in the method section, please check the Line 464-468.

14. Why the haplotypes with less than six rice accessions were remove from Figure 4f? It should be explanted.

Response: Many thanks for your suggestion. In order to illustrate the origin and evolution of functional types of *OsGluA2*, the haplotype network was constructed using the haplotypes with more than six accessions. We have eliminated the rare haplotypes in this analysis because of two reasons. Firstly, the appearance of rare alleles could potentially be attributed to sequencing errors. Secondly, the rare haplotypes could influence the intelligibility of haplotype network, because many haplotypes only contain one or very few accessions. When we tried to detect the haplotypes using all the accessions, a total of 87 haplotypes were detected, and most of them were carried by one or two accessions. Thus, a haplotype network using all these 87 haplotypes will be unintelligibility in illustrating their evolutionary relationship. More attention, the major 15 haplotypes in this analysis cover more than 94.5% of the total accessions.

According to your suggestion, we used all the polymorphic sites with minor allele frequency ≥0.005 in the *OsGluA2* locus to construct the phylogenetic tree and haplotype network, and a total of 87 haplotypes were identified. Moreover, the analysis of haplotype network have used all these 87 haplotypes in the revised manuscript (**Fig. 5e** and **5f** in the revised manuscript).

15. It is hard to understand the sentence in line 420-422. It needs to be revised.

Response: Thank you for pointing it out. Following your suggestion, we have reworded the sentence in line 420-422, please check it.

16. In line 478, LGC-1 mutant and W3660 were mentioned. However, the glutelin contents of these cultivars were not provided.

Response: Thank you for pointing it out. Following your suggestion, we have added the glutelin contents of LGC-1 mutant and W3660 in Line 444.

17. Some of the journal names in the reference were full name, but the others were abbreviated name.

Response: We very much appreciate your careful reading of our manuscript, and it has been corrected in the revised manuscript.

18. 'FLOURY ENDOSPERM2' in reference 19 (line 656) should be italic.

Response: Sorry for the mistake, and it has been corrected in the revised manuscript.

19. Page number of reference 12 (line 641-642) was not provided.

Response: Sorry for the mistake, and it has been corrected in the revised manuscript.

20. The comma was missing in '16 000rpm' in line 572. In addition, one space was also missing.

Response: Sorry for the mistake, and it has been corrected in the revised manuscript.

Reviewer #3 (Remarks to the Author):

This paper reports the identification of a locus, Gpc-10, that influences grain protein content. The authors show that the locus encodes a glutelin, OsGlu-A2 that is generally expressed more highly in the Indica subspecies than Japonica. CRISPR knockouts and over-expression are used to show that the expression of the gene does influence grain protein content. The authors provide comprehensive data.

I feel that the authors have uncovered the hints of an intriguing story. Based on their results and without preconceptions of what the OsGlu-A2 gene does, it would seem that this is like a transcription factor/RNA regulator that up-regulates the transcription of a number of storage protein genes. The authors point out (lines 228-231), the increased protein content is not just due to increased accumulation of OsGlu-A2 itself but generalized increase of protein accumulation. So somehow this increased expression of OsGlu-A2 is triggering wide-spread change in protein expression. Some of the changes in the transcription is far greater than shown by OsGlu-A2 itself. For example, the transcription of Os-GluA1 according to Fig 2d is increased about three fold.

Response: Thanks very much for your positive comments. As for your guess, we conducted analysis for the small RNA, however, we failed to detect some small RNA target to *OsGluA2,* suggesting that, in the 35 Kb region, there is no small RNA which could

regulate the glutelin synthesis genes (details see below). In fact, accumulated knowledge showed that the storage proteins can be coordinately increase or increase in the knock-down transgenic lines. Please see the evidence in the Discussion section.

I would suggest the authors consider the following to make the story clearer.

1. Is it possible that some small RNAs are being transcribed from this same region that is influencing transcription of a large number of genes? In that case the OsGlu-A2 gene is almost a by-stander. Is it possible to do a sequence comparison of the small RNAs in the paired knockout and over-expressing lines?

Response: Many thanks for your suggestion. We fully agree that your suggestions are worth to try. In view of the special growth circle in rice, it is difficult to obtain RNAs of rice grain in winter. Considering the timeliness of the research article, we will seriously take your advices and consider the evidences in the future studies. However, based on your suggestion, we analysis the genomic sequence of *OsGluA2* by using the data of small RNAs in developing rice grains (Zhu, Q. et al. 2008). The result showed that no small RNAs were generated in the promoter region and coding region of *OsGluA2* (**Fig.1**). At the same time, we further predicted small RNAs around the SNPs between our two parents (http://www.mirbase.org/). The result showed that no matches were found for our nucleotide sequence. In our work, we successfully narrowed the *qPC-10* into a 35 Kb region by map-based cloning. The gene function of *OsGluA2* was validated by genetic complementary transformation and CRISPR-Cas9. By analyzing *OsGluA2* variations in 100 rice accessions and conducting transient assays, we concluded that SNP - 1100 might be functional SNP (FNP) accounting for the expression level differences between the *OsGluA2* alleles and GPC variance. So, we have reasons to believe that *OsGluA2* is the candidate gene in our research.

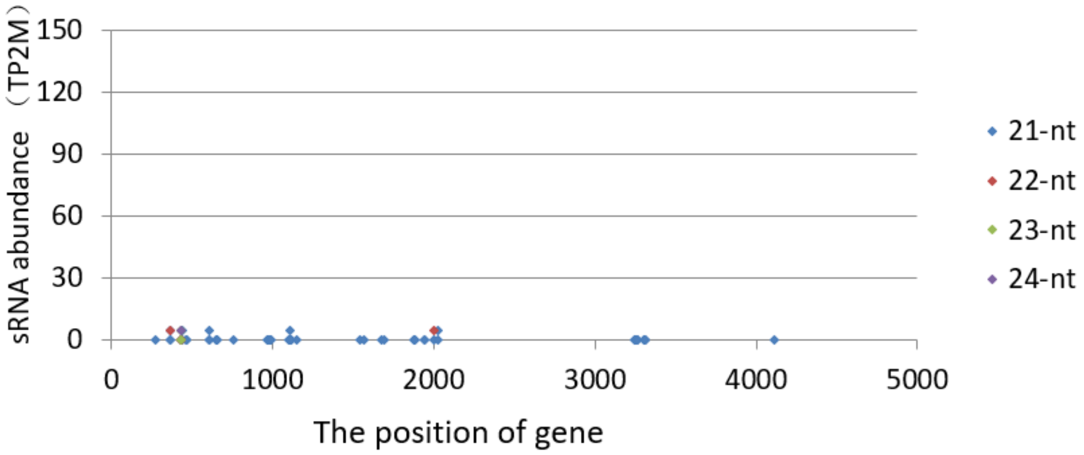

Fig.1 The detection of sRNA of *OsGluA2* in 1-5 DAF and 6-10 DAF grains. The number 1 and 5000 in x-coordinate represent the position 13497239 and 13501435 in Chr.10.

Zhu, Q. *et al*. A diverse set of microRNAs and microRNA-like small RNAs in developing rice grains. *Genome Res*. **18**, 1456-1465 (2008).

2. The paper does not directly show how much greater OsGlu-A2 protein accumulation is. There is the data on the expression at the RNA level but no qualitative or quantitative assay of OsGlu-A2 protein. Is it in fact accumulating more? Is it not possible to use antibodies/Western blotting to show directly that the increased transcription of the RNA is leading to more of the OsGlu-A2 protein accumulation? The results from this may indicate mechanisms by which the general increase is taking place.

Response: Thanks very much for your comments. Glutelins are encoded by a small multigene family of about 12 genes per haploid genome. These genes are divided into four subfamilies, designated *GluA, GluB, GluC* and *GluD*. The GluA and GluB members share 60% to 65% homology in coding sequence, whereas members within the same subfamily share more than 80% homology (GluA1, 2 and 3) (Kawakatsu, 2010). Due to the fact, it is impossible to generate the antibody specific to *OsGluA2*. Only OsGluA antibody is available, by which we cannot differentiate the members of GluA.

Kawakatsu, T, & Takaiwa, F. Cereal seed storage protein synthesis: fundamental processes for recombinant protein production in cereal grains. *Plant Biotechnol J.* **8**, 939–953 (2010).

Minor points

1. In general the paper reads well although some editing is required of the English. For example it should be ‘chose’ not ‘choose’ in line 198 and some other sentence constructions (starting with And etc) left me a little uncomfortable.

Response: We very much appreciate your careful reading of our manuscript. We have checked the MS carefully and removed the “and” in the revised MS.

2. In Fig 2c the colouring is wrong and reverse of what it should be.

Response: Sorry for the mistake, and it has been corrected in the revised manuscript.

3. It would be good to have a Table with list of the names of the genes being assayed in Fig2d. This could go in the supplementary data. For example I cannot immediately find out what SMP is.

Response: Thank you for pointing it out. Following your suggestion, we have added the names of the genes being assayed in **Fig.3d** and the function description in the supplementary data (**Supplementary Table 12** in the revised manuscript), please check it.

Reviewers' comments:

Reviewer #1 (Remarks to the Author):

Major points:
Revisions were well done and my comments raised to the previous version were almost solved and were reflected to the revised one. I believe the revised version is acceptable to publish except only one point. I now understand the difference between qPC1 reported by Peng (ref.35) and qPC-1 and -10 in the current MS is only in the absence/presence of hyphen, very tiny letter. I strongly suggest the authors to re-name the QTL identified in this work as qGPC (grain protein content) to avoid confusion among rice research community. The reviewer is at the neutral site, not at qPC1 site. In general, naming in the earlier publication should be respected.

Minor points:
Line 68, 57 KD should read 57 kD (in case of protein, kilo Dalton is generally abbreviated as kD, not KD).
Line 69, 39 KD and 23 KD should read 39 kD and 23 kD
Line 246, 17-KD should read 17-kD
Line 396, 397 & 398: 13-KD prolamins should read 13-kD prolamins.
Line 418: Why nutrition quality? Is this to be an eating quality or eating preference?
Line 424: Is the expression "decrease in nutritional quality" appropriate as in Line 418?
Line 429-432: Two sentences continued only with comma. Please use some adverb to combine these two sentences.
Supple.Fig.1c, KD should read kD

Reviewer #2 (Remarks to the Author):

Most of the questions and concerns have been well addressed. There are now two remaining problems:
1. The evidences on the causative variation on the OsGluA2 promoter seem not be solid. More experiments and analyses are needed.
2. It is not true that "basmati rice belong to indica subspecies". The phylogenetic trees through whole-genome resequencing (see 3k rice genomes, Wang et al Nature 2018; 66 representative rice lines by Zhao et al Nature Genetics 2018) have showed that basmati is genetically close to japonica subspecies.

Reviewer #3 (Remarks to the Author):

I attach an annotated version of the manuscript. The comments are minor and aimed at improvement of the reading of the manuscript. Some sections of the manuscript do not have many comments but that is probably because I was not reading those sections very carefully.

I do not have any scientific criticisms of the manuscript. It represents an enormous amount of work. The English is also generally good but there are small improvements that can be made. I have suggested some of them. It is possible that the authors will find other places for improvement by careful re-reading of the manuscript.

Response to Referees

Reviewers' comments:

Reviewer #1 (Remarks to the Author):

Major points:

Revisions were well done and my comments raised to the previous version were almost solved and were reflected to the revised one. I believe the revised version is acceptable to publish except only one point. I now understand the difference between qPC1 reported by Peng (ref.35) and qPC-1 and -10 in the current MS is only in the absence/presence of hyphen, very tiny letter. I strongly suggest the authors to re-name the QTL identified in this work as qGPC (grain protein content) to avoid confusion among rice research community. The reviewer is at the neutral site, not at qPC1 site. In general, naming in the earlier publication should be respected.

Response: Thank you for underlining this deficiency. We totally agree with your opinion and therefore have renamed the QTL identified in this work as qGPC to avoid confusions among rice research community. Please check it in the revised manuscript.

Minor points:

Line 68, 57 KD should read 57 kD (in case of protein, kilo Dalton is generally abbreviated as kD, not KD).

Line 69, 39 KD and 23 KD should read 39 kD and 23 kD

Line 246, 17-KD should read 17-kD

Line 396, 397 & 398: 13-KD prolamins should read 13-kD prolamins.

Supple.Fig.1c, KD should read kD

Response: We very much appreciate your careful reading of our manuscript. Following your suggestions, the mistakes have been corrected in the revised manuscript, please check it.

Line 418: Why nutrition quality? Is this to be an eating quality or eating preference?

Response: Thanks for your comments. Many researches showed that GPC is inversely correlated with palatability. High GPC may lead to densely structured rice grains, which will result in hard and loose cooked rice and, thus poor palatability. Therefore, if the *OsGluA2*^{LET} allele from *Japonica* accessions is directly replaced with *OsGluA2*^{HET}allele, the GPC of *Japonica* accessions will be increased and the nutrition quality will be increased consequently.

Line 424: Is the expression "decrease in nutritional quality" appropriate as in Line 418?

Response: Thanks for your comments. We previously identified a major QTL *qPC-1* (ref. 28). We revealed that the allele from Habataki can significantly decrease the glutelin content, consequently leading to the decrease in GPC and nutritional quality. In order to resolve the confusion, we have reworded the sentence, please check line 516.

Line 429-432: Two sentences continued only with comma. Please use some adverb to combine these two sentences.

Response: Thank you for pointing it out. Following your suggestion, we have reworded the sentence to "In addition, some landrace or cultivars (LGC-1 mutant and W3660) with low glutelin contents (less than about 20% of the total GPC) are identified and have been served as gene resource to develop new cultivars, which are suitable for patients affected with diabetes and kidney failure.".

Reviewer #2 (Remarks to the Author):

Most of the questions and concerns have been well addressed. There are now two remaining problems:

1. The evidences on the causative variation on the OsGluA2 promoter seem not be solid. More experiments and analyses are needed.

Response: Thank you very much for your comments. For making the evidence on the causative variation be more solid, more independent transformations (n=10) for each sample were performed, and the transfection system was further optimized (half of RISBZ1 transcription factor dosage used in this assay). The result once again demonstrated that A/T mutation at -1100 (M3) plays a crucial role in determining the promoter transcription efficiency. The relative activities of M3 was greatly increased ($P \leq 0.01$), close to SL431; whereas, M1, M2 and M4 exhibited low transcription activities at same level of Sasanishiki (Sa), significantly lower than M3 and SL431 (**Fig. 4f**). At the same time, we totally understand that the CRISPR or other transgenic experiments on the promoter may better define the causative variants. In view of the special growth circle in rice, at least one year will be needed. Considering the timeliness of the research article, we will seriously take your advices and consider the evidences in the future studies.

2. It is not true that "basmati rice belongs to indica subspecies". The phylogenetic trees through whole-genome resequencing (see 3k rice genomes, Wang et al Nature 2018; 66 representative rice lines by Zhao et al Nature Genetics 2018) have showed that basmati is genetically close to japonica subspecies.

Response: Thank you very much for underlining this deficiency. After carefully reading the latest literatures following your directions, we now totally agree with your point that "basmati is genetically close to *Japonica* subspecies". Following your suggestions, we clarified Basmati rice into japonica group and reanalyzed the data in the revised manuscript, please check it.

Reviewer #3 (Remarks to the Author):

I attach an annotated version of the manuscript. The comments are minor and aimed at improvement of the reading of the manuscript. Some sections of the manuscript do not have many

comments but that is probably because I was not reading those sections very carefully.

I do not have any scientific criticisms of the manuscript. It represents an enormous amount of work. The English is also generally good but there are small improvements that can be made. I have suggested some of them. It is possible that the authors will find other places for improvement by careful re-reading of the manuscript.

Response: Thank you very much for your comments. Following your suggestions, we have improved the English and revised the manuscript to better describe our points and to clarify possible confusions, please check it.

REVIEWERS' COMMENTS:

Reviewer #1 (Remarks to the Author):

Revisions were well done. After changing the name of QTL from qPC to qGPC, your new QTL gained independency from the previous publication.

Reviewer #2 (Remarks to the Author):

I'm satisfied with the revision and have no further questions.

Reviewer #1 (Remarks to the Author):

Revisions were well done. After changing the name of QTL from qPC to qGPC, your new QTL gained independency from the previous publication.
Response: Thank you very much.

Reviewer #2 (Remarks to the Author):

I'm satisfied with the revision and have no further questions.
Response: Thank you very much.
